# Epitaxial CsPbBr_3_/CdS Janus Nanocrystal Heterostructures for Efficient Charge Separation

**DOI:** 10.1002/advs.202206560

**Published:** 2023-02-25

**Authors:** Hengwei Qiu, Fu Li, Shan He, Ran Shi, Yaoyao Han, Hannikezi Abudukeremu, Lin Zhang, Yan Zhang, Song Wang, Wangyu Liu, Chao Ma, Honghua Fang, Run Long, Kaifeng Wu, Hao Zhang, Jinghong Li

**Affiliations:** ^1^ Department of Chemistry Center for BioAnalytical Chemistry Key Laboratory of Bioorganic Phosphorus Chemistry & Chemical Biology of Ministry of Education Tsinghua University Beijing 100084 China; ^2^ State Key Laboratory of Molecular Reaction Dynamics Dalian Institute of Chemical Physics Chinese Academy of Sciences Dalian 116023 China; ^3^ University of the Chinese Academy of Sciences Beijing 100049 China; ^4^ College of Chemistry Key Laboratory of Theoretical & Computational Photochemistry of Ministry of Education Beijing Normal University Beijing 100875 China; ^5^ Department of Precision Instrument State Key Laboratory of Precision Measurement Technology & Instruments Tsinghua University Beijing 100084 China

**Keywords:** colloidal nanocrystals, epitaxial heterostructures, perovskites, photoconductors, quantum dots

## Abstract

Epitaxial heterostructures of colloidal lead halide perovskite nanocrystals (NCs) with other semiconductors, especially the technologically important metal chalcogenides, can offer an unprecedented level of control in wavefunction design and exciton/charge carrier engineering. These NC heterostructures are ideal material platforms for efficient optoelectronics and other applications. Existing methods, however, can only yield heterostructures with random connections and distributions of the two components. The lack of epitaxial relation and uniform geometry hinders the structure–function correlation and impedes the electronic coupling at the heterointerface. This work reports the synthesis of uniform, epitaxially grown CsPbBr_3_/CdS Janus NC heterostructures with ultrafast charge separation across the electronically coupled interface. Each Janus NC contains a CdS domain that grows exclusively on a single {220} facet of CsPbBr_3_ NCs. Varying reaction parameters allows for precise control in the sizes of each domain and readily modulates the optical properties of Janus NCs. Transient absorption measurements and modeling results reveal a type II band alignment, where photoexcited electrons rapidly transfer (within ≈9 picoseconds) from CsPbBr_3_ to CdS. The promoted charge separation and extraction in epitaxial Janus NCs leads to photoconductors with drastically improved (approximately three orders of magnitude) responsivity and detectivity, which is promising for ultrasensitive photodetection.

## Graphical abstract

CsPbBr_3_/CdS Janus nanocrystals with evident epitaxial relation and adjustable sizes, previously inaccessible for perovskite/metal chalcogenide nanocrystal heterostructures, are synthesized. The strongly coupled, type II alignment enables the ultrafast transfer of photoexcited electrons (within ≈9 ps) from CsPbBr_3_ to CdS domain, leading to over three orders of magnitude increases in photoconducting behaviors.

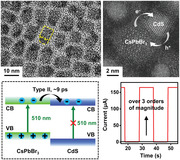

## 1 Introduction

Colloidal lead halide perovskite nanocrystals (NCs), such as the inorganic CsPbX_3_ (X = Cl, Br, I), have emerged as a promising semiconductor material platform for widespread technological applications.^[^ ^1^, ^2^, ^3^, ^4^, ^5^, ^6^ ^]^ For instance, perovskite NC‐based light‐emitting diodes (LEDs),^[^ ^7^, ^8^ ^]^ solar cells,^[^ ^9^, ^10^ ^]^ and X‐ray/photodetectors^[^ ^11^, ^12^ ^]^ have shown performance rivaling or even outperforming those of traditional semiconductor NCs (or quantum dots, QDs) and organic materials. This follows from the rapid development of synthetic routes for perovskite NCs with well‐defined sizes/shapes/surface chemistries and associated properties, such as the high emission efficiency and large charge carrier mobilities, which are central to photoelectric/photochemical applications.

Beyond single‐component perovskites, NC heterostructures of perovskites with other functional materials have been a new design paradigm toward advanced material properties and device performance.^[^ ^13^, ^14^, ^15^ ^]^ One of the most desirable cases is the epitaxial heterostructures coupling a perovskite domain with a domain of another semiconductor NC, especially those based on metal chalcogenides. Epitaxial heterostructures, which involve the atomically aligned growth of a crystalline semiconductor atop another, have been the workhorse for both conventional semiconductor industry and colloidal semiconductor QDs. In these well‐established systems, the epitaxial interactions introduce strong coupling between the constituents and minimize defects at the interface. This provides advanced functionalities in surface passivation and wavefunction engineering, leading to better controllability in carrier recombination or separation tailored for different applications.^[^ ^16^, ^17^, ^18^, ^19^ ^]^ In analogy, proper combinations of perovskite and metal chalcogenide NCs are also anticipated to produce epitaxial heterostructures that can create new photophysical properties and unprecedentedly high device performance. For example, epitaxial perovskite NC heterostructures with type I band alignments (i.e., the conduction and valance bands of perovskites are within those of metal chalcogenides), though not synthesized yet, are proposed as the best solution for addressing the stability issues of perovskite LEDs.^[^ ^20^ ^]^ On the other hand, type II epitaxial heterostructures (i.e., with staggered conduction and valance bands) are promising in breaking the Shockley–Queisser limit in perovskite photovoltaics,^[^ ^21^, ^22^ ^]^ and also in reducing the undesired charge carrier recombination in perovskite NC‐based photodetectors.^[^ ^21^, ^23^ ^]^

Unfortunately, the synthesis of epitaxial colloidal NC heterostructures of perovskites with other semiconductors, especially metal chalcogenides, has not been accomplished.^[^ ^24^ ^]^ As accounted in several recent perspectives and reviews,^[^ ^14^, ^15^, ^20^, ^25^ ^]^ there are only a limited number of reports on the heterostructures of CsPbX_3_ NCs with CdS,^[^ ^26^, ^27^ ^]^ ZnS,^[^ ^28^, ^29^ ^]^ PbS,^[^ ^30^, ^31^ ^]^ and PbSe^[^ ^32^ ^]^ in nonuniform core/shell and other geometries. But none of them show evidence of facet–facet orientation or epitaxial relation between the two components. Meanwhile, the obtained heterostructures mostly entail random deposition of islands/clusters (with limited control over the sizes or numbers) or discontinuous shelling of metal chalcogenides on polydispersed CsPbX_3_ NCs, and sometimes also contain impurity phases.^[^ ^24^ ^]^ The unsatisfactory morphological control complicates the correlation between the structural design and the photophysical properties of the heterostructures. Moreover, these randomly connected, nonepitaxial interfaces generally involve dangling bonds and trap states that are unfavorable for both the radiative charge combination and the interfacial electron transport, sacrificing their performance in optoelectronics or photoelectric/chemical conversions.^[^ ^33^ ^]^

The main challenge for synthesizing such epitaxial NC heterostructures lies in the large discrepancy in the formation energies of the ionic perovskites and covalent metal chalcogenides.^[^ ^24^ ^]^ The growth of metal chalcogenide NCs on perovskites thus faces a thermodynamically high energetic barrier and a stark contrast in the formation kinetics (fast for perovskites and slow for metal chalcogenides). Existing methods either use presynthesized CsPbX_3_ NCs as seeds for metal chalcogenides nucleation or depend on simultaneous growth of the two components from molecular precursors; both suffer from the imbalanced growth thermodynamics/kinetics of the two domains.

In this work, we report the first synthesis of epitaxial perovskite/metal chalcogenide (CsPbBr_3_/CdS) Janus NC heterostructures with precisely tunable morphological and photophysical properties, featuring ultrafast charge separation at the electronically coupled interface. The key to the synthesis is the use of preformed nanoclusters as a single‐source precursor for CsPbBr_3_, which accommodates the large discrepancies in the formation kinetics of the two components. The obtained Janus heterostructures contain a hemispherical CdS domain grown exclusively on one facet of the cuboid CsPbBr_3_ NCs. The epitaxial relation between CsPbBr_3_(220) and CdS(220) facets (lattice mismatch smaller than 1.0%) is confirmed by both microscopic images and density function theory (DFT) calculations. Varying temperatures and other reaction parameters allow for the exquisite control of sizes of each component (from ≈5 to ≈20 nm with narrow size distributions), leading to readily tunable optical properties. Transient absorption (TA) measurements reveal a type II band alignment, where the strongly coupled heterojunction permits an ultrafast electron transfer (within ≈9 ps) from CsPbBr_3_ to CdS domain. Such rapid charge separation reduces the energy losses in photoelectric conversion and translates to significantly improved photoconducting behaviors. Janus NC‐based photoconductors show responsivity (11.2 A W^−1^) and detectivity significantly higher than those achievable for pure CsPbBr_3_ NCs, by approximately three orders of magnitude. The syntheses can also be extended to the combinations of perovskite materials containing different halides (CsPbX_3_/CdS, X = Cl and I) and other metal chalcogenides (e.g., CsPbBr_3_/ZnS). These epitaxial NC heterostructures with structurally and electronically coupled domains can considerably expand the material toolbox of perovskite NCs with extended optical and electronic functionalities.

## 2 Results and Discussion

### 2.1 Synthesis of Epitaxial CsPbBr_3_/CdS Janus NCs

The synthesis used preformed CsPbBr_3_ nanoclusters^[^ ^34^, ^35^, ^36^, ^37^ ^]^ as precursors for perovskites, and cadmium oleate (Cd(OA)_2_) and sulfur in 1‐octadecene (S‐ODE) as precursors for CdS. Details appear in the Supporting Information. For all the synthesis, we used CsPbBr_3_ nanoclusters with the excitonic peak at ≈400 nm. In brief (Figure S1, Supporting Information), a solution of Cd(OA)_2_ and 1‐dodecanethiol (DDT) was added in a flask containing preheated ODE (150–220 °C) under nitrogen, followed by the swift injection of a solution of CsPbBr_3_ nanoclusters and S‐ODE. The reaction was quenched after a certain time (e.g., 3 min). Janus NCs were then collected by centrifugation, purified with hexane/methyl acetate as solvent/nonsolvent, and redispersed in nonpolar solvents such as hexane and toluene.

**Figure 1:**
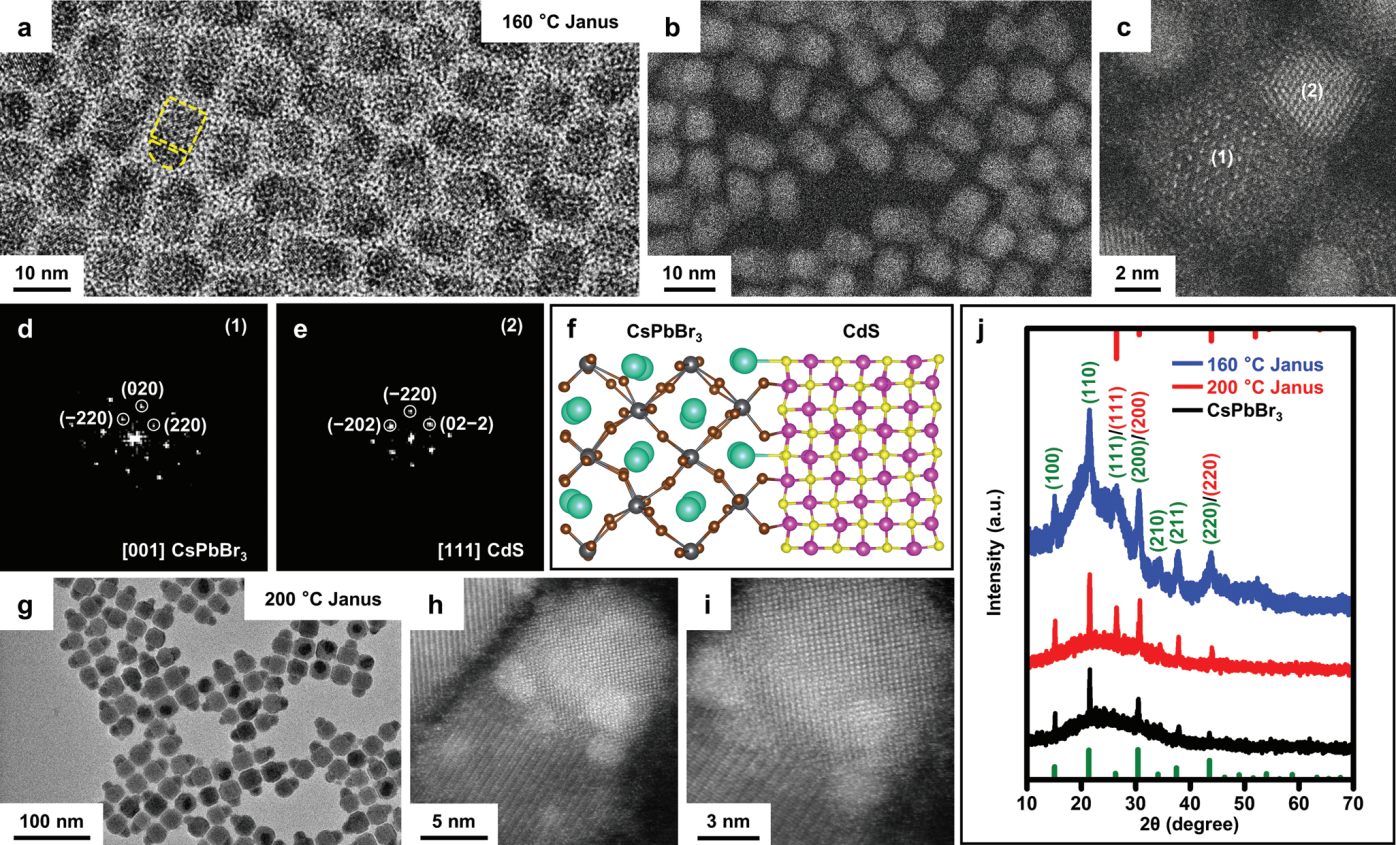
Structural analysis of epitaxial CsPbBr_3_/CdS Janus NCs. a) TEM and b,c) STEM images of Janus NCs synthesized at 160 °C. The yellow dashed shapes in (a) indicate the joint CsPbBr_3_ cuboid and CdS hemisphere. d,e) FFT analysis of domains 1 (CsPbBr_3_) and 2 (CdS) in the single Janus NCs shown in (c). f) A scheme of the structural model of the alignment of CdS {220} plane and CsPbBr_3_ {220} plane along [220]. Colors of atoms: Cs (green), Pb (gray), Br (brown), Cd (magenta), and S (yellow). g) TEM and h,i) STEM images of Janus NCs synthesized at 200 °C. j) XRD patterns of Janus NCs synthesized at 160 and 200 °C, respectively, and plain CsPbBr_3_ NCs (edge length, 16 nm). The vertical lines are standard diffraction peaks for orthorhombic CsPbBr_3_ (bottom, green) and zinc blende CdS (top, red) phases. The numbers in parentheses indicate the indices of corresponding diffraction peaks (green, CsPbBr_3_; red, CdS).

Transmission electron microscopic (TEM) images of NCs synthesized at 160 °C (**Figure** **1**a and Figure S1, Supporting Information) show uniform Janus heterostructures of nanoscale CsPbBr_3_ cuboids coupled with hemispherical CdS domains exclusively on one of the six facets. The magnified high angle annular dark field scanning transmission electron microscopic (HAADF‐STEM) images (Figure 1b) and related statistical analysis on over 100 NCs confirm the uniform edge length of CsPbBr_3_ domains (5.5 ± 1.1 nm), which also dictates the diameter of the CdS hemispheres. We measured the composition of the CdS domains by using inductively coupled plasma‐optical emission spectroscopy, showing a Cd/S/Pb ratio (1:0.997:0.008) consistent with the CdS stoichiometry. STEM image of a typical, single Janus NC (Figure 1c) confirms the two single‐crystalline domains; the CsPbBr_3_ domain is in orthorhombic perovskite phase while the CdS domain is in cubic zinc blende phase. Based on the fast Fourier transform (FFT) analysis of the NC shown in Figure 1c, the CsPbBr_3_ domain is facing the [001] zone axis, exposing {220} and {020} planes (Figure 1d and Figure S2, Supporting Information). The CdS domain is in the [111] orientation and features {220} planes (Figure 1e). This suggests the interface is composed of CdS (−202) planes grown on CsPbBr_3_ (220) planes. In real space, these two planes share similar lattice constants with negligible mismatch (<1.0%, *d*_CsPbBr3_{220} = 2.0610 Å, *d*_CdS_{220} = 2.0614 Å). The angles between (−220) and [001] of CsPbBr_3_ are the same as that between (−211) and [111] of CdS. Hence, the orientation relations are identified as {220}CsPbBr_3_ || {211}CdS; [001]CsPbBr_3_ || [111]CdS. Figure 1f further depicts the heteroepitaxy formed across the interface, highlighting the atomically coherent alignment of the {220}CsPbBr_3_ and {220}CdS planes. To the best of our knowledge, this is the first observation of epitaxial interface in perovskite NC heterostructures with metal chalcogenides, although recent work revealed epitaxial structures composed of CsPbX_3_ and Pb_4_S_3_Br_2_ chalcohalides.^[^ ^34^, ^38^ ^]^ Heterostructures in previous reports do not show epitaxial junction due to a combination of poor size/shape control, ambiguous shell formation, and impurity phases.^[^ ^14^ ^]^ The epitaxial growth of highly crystalline domains in our Janus NCs probably benefit from the relatively high reaction temperatures that are imperative for the growth of covalent CdS.

Higher reaction temperatures (e.g., 200 °C) led to Janus NCs with larger sizes in both domains but similar size uniformity. CdS hemispheres still exclusively grow on a single facet of CsPbBr_3_ nanocubes (average edge length, 16.4 ± 2.5 nm, Figure 1g and Figure S1c, Supporting Information). High‐resolution STEM images of a single Janus NC (Figure 1h,i) clearly show the different planes across the interface. We also performed the synthesis at 200 °C in the absence of Cd(OA)_2_ and S‐ODE. Only were plain cuboid CsPbBr_3_ NCs obtained (16.1 ± 2.1 nm, denoted as 16 nm CsPbBr_3_ NCs in the following discussions for simplicity) (Figure S1d, Supporting Information), in line with previous reports on the synthesis of CsPbBr_3_ NCs using preformed clusters.^[^ ^36^ ^]^ The powder X‐ray diffraction (XRD) patterns of Janus NCs synthesized at 160 and 200 °C (Figure 1j and Figure S3, Supporting Information) contain features from both components. Characteristic diffraction peaks/peak shifts for zinc blende CdS were observed, including (111) (≈26°), (200) (≈31°), and (220) (≈43°). Figure S3 in the Supporting Information is the magnified view of XRD patterns, highlighting these CdS‐related peaks.

The morphology of CsPbBr_3_/CdS Janus NCs can be precisely controlled by using various reaction parameters. The most imperative parameter for our synthesis is the choice of preformed nanoclusters as CsPbBr_3_ precursors. Nanoclusters have been used as single‐source precursors for the seeded growth of perovskites in different dimensions^[^ ^36^ ^]^ and with complex geometries.^[^ ^39^ ^]^ The synthesis of nanoclusters was first reported by Pradhan group,^[^ ^36^ ^]^ where the nanocluster sizes can be tuned by the reaction time. CsPbBr_3_ NCs prepared from such nanoclusters show high structural stability when heated in the presence of solvents/ligands. In comparison, NCs formed from pyrolysis of molecular precursors suffer from overgrowth/sintering or disintegration at similar or even lower temperatures. In the synthesis of Janus NCs, the structural stability of CsPbBr_3_ nanoclusters cannot only slow down the growth kinetics of perovskite domains, but also facilitate the growth of CdS domains at higher temperatures to overcome the energetic barrier. This enables the formation of well‐defined CdS domains at high reaction temperatures without degrading the perovskite domains, outperforming those achieved in previous attempts. We also attempted to synthesize Janus NCs with preformed CsPbBr_3_ NCs as seeds for CdS growth. Only random Janus structures with large particle sizes and wide size distribution were achieved (Figure S4, Supporting Information). Interestingly, the sizes of CsPbBr_3_ nanoclusters are also important (Figure S5, Supporting Information). For the synthesis of Janus NCs, we used nanoclusters with the first excitonic peak at ≈400 nm. Nanoclusters with larger sizes yielded Janus NCs with inferior quality, such as the inhomogenous shapes and size distribution (Figure S5, Supporting Information). The size of nanoclusters with the first excitonic peak at ≈400 nm was initially estimated to be ≈0.6 nm by using the empirical equation for spherical QDs.^[^ ^36^ ^]^ A recent work by Manna and co‐workers revealed that these CsPbBr_3_ nanoclusters are ≈2 nm thick nanodisks (diameter ≈13 nm) with Br‐deficient composition and distorted orthorhombic structures, based on results from X‐ray pair distribution function measurements.^[^ ^37^ ^]^ Nanoclusters with the first excitonic peak at ≈400 nm (and presumably with disk‐shape according to ref. [36]) are optimal for the synthesis.

**Figure 2:**
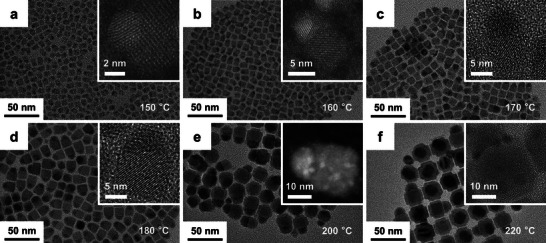
Effect of reaction temperatures on the synthesis of Janus NCs. a–f) TEM images of samples synthesized at different temperatures, ranging from 150 to 220 °C. The insets show the magnified view of a single Janus NC. In all cases, CdS hemispheres grow exclusively on a single facet of CsPbBr_3_.

Uniform Janus NCs can be synthesized in a wide temperature window from 150 to 220 °C (**Figure** **2**a–f and Figure S6, Supporting Information). Insets in Figure 2 show representative individual Janus NCs. The sizes of both CdS and CsPbBr_3_ increase with elevated temperatures while their size/shape uniformity largely maintain. For instance, Janus NCs synthesized at 150 and 160 °C contain CsPbBr_3_ and CdS with sizes in the quantum confinement regime while those synthesized above 180 °C are composed of weakly quantum‐confined domains. The CsPbBr_3_ domain for Janus NCs synthesized at 220 °C evolve into armed/hexapod structures with more exposed {110} and {001} facets. This shape transition is in line with those observed for CsPbBr_3_ NCs synthesized from nanocluster precursors in the presence of oleylammonium bromide at similar temperatures.^[^ ^39^ ^]^

We monitored the growth process of Janus NCs by varying the reaction time. Immediately (<10 s) after the injection, CsPbBr_3_ nanoclusters transform into cuboid NCs that largely maintain their sizes in the following reactions (Figures S7–S9, Supporting Information). This corroborates the assumption that NCs synthesized from CsPbBr_3_ nanoclusters have high structural stability and slow growth kinetics. Small clusters of (presumably) CdS appear in the course of 30 s to 1 min at 160 °C and randomly locate at the edges/corners of predominantly one facet of CsPbBr_3_. The formation of evident CdS hemispheres occurs after 2–3 min, yielding well‐defined Janus heterostructures. Similar trend was observed for samples synthesized at 200 °C. For all syntheses, CdS grow solely on one facet of CsPbBr_3_ domains. This may be related to the slow nucleation kinetics of CdS due to their highly covalent bond characters. As soon as the nucleus of CdS forms on one facet, the heteroepitaxial interactions reduce the energy barrier for further growth on the same facet. Similar Janus heterostructures were also observed in the epitaxial CsPbX_3_/Pb_4_S_3_Br_2_ NCs, where Pb_4_S_3_Br_2_ dominantly grow on a single facet of CsPbBr_3_.^[^ ^34^, ^38^ ^]^

In parallel, S precursors also affect the synthesis. The contrast in the formation energy of ionic CsPbBr_3_ and covalent CdS requires highly reactive S precursors to compensate the slow kinetics in CdS nucleation and growth. S‐ODE has moderate reactivity and can only afford NCs with random, tiny CdS domains and poor shape/size homogeneity (Figure S10, Supporting Information). Adding a small amount of DDT (e.g., DDT:S ≈ 1:100 in molar ratio, Figure S10b, Supporting Information) significantly increases the sizes of CdS and improves the quality of obtained Janus NCs. The role of DDT in promoting the nucleation and growth of CdS was supported by UV‐visible absorption spectra in Figure S11 in the Supporting Information. In the absence of DDT, the reaction of Cd(OA)_2_ and S‐ODE produces small CdS NCs with narrow absorption characteristics below 400 nm. Adding DDT to the synthesis yields larger CdS nanoparticles with the first excitonic peak at ≈450 nm. Other S sources such as S‐oleylamine can also be used for synthesizing CsPbBr_3_/CdS Janus NCs, although with lower size/shape uniformity due to their lower reactivity (Figure S12, Supporting Information).

### 2.2 Optical Properties of Janus NCs and the Rapid Charge Transfer across the Type II Heterostructures

**Figure 3:**
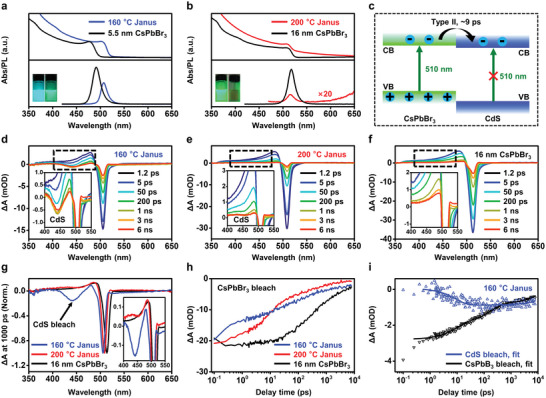
Steady‐state and transient optical spectra of Janus NCs. a,b) Steady‐state UV‐visible absorption (top) and PL (bottom) spectra of Janus NCs synthesized at different temperatures (a, 160 °C and b, 200 °C) and corresponding control samples (i.e., CsPbBr_3_ NCs with the same edge lengths with the CsPbBr_3_ domains in Janus NCs). The PL intensity of Janus NCs synthesized at 200 °C (bottom in b) is magnified by 20 times for clarity. Insets in (a,b) show the photos of NC solutions (left, CsPbBr_3_ NCs; right, Janus NCs) under UV light. c) Schematic type II band alignment in the epitaxial Janus NCs. Under selective excitation (e.g., 510 nm for the CsPbBr_3_ domains), photogenerated electrons transfer to CdS domain while holes are mostly confined in CsPbBr_3_. d–f) TA spectra of Janus NCs synthesized at d) 160 and e) 200 °C and f) 16 nm CsPbBr_3_ NCs, at a pump wavelength of 510 nm. g) Comparison of TA spectra of these samples at 1000 ps, highlighting the bleach of CdS excitons due to the electron transfer process in Janus NCs. The spectra were normalized based on the ΔA related to CsPbBr_3_ bleach (≈500 nm). h) The CsPbBr_3_ exciton bleach recovery kinetics of samples. i) Comparison of the kinetics of CsPbBr_3_ bleach decay (black triangles) and CdS bleach formation (blue triangles) in Janus NCs synthesized at 160 °C, and related fits (lines).

The precise synthetic control of epitaxial Janus NCs allows for exploring their structure‐dependent optical properties. **Figure** **3**a,b shows the steady‐state optical absorption and PL spectra of Janus NCs synthesized at 160 and 200 °C, respectively. For comparison, we prepared CsPbBr_3_ NCs with the same edge lengths of the CsPbBr_3_ domains in corresponding Janus NCs (Figures S13–S15 and Table S1, Supporting Information). Janus NCs synthesized at 160 °C show red‐shifted absorption and PL spectra relative to the control samples (5.5 nm CsPbBr_3_ NCs). This leads to their different emission colors (blue for CsPbBr_3_ and green for Janus NCs, inset in Figure 3a and Figure S16, Supporting Information). Additionally, the Janus NCs show increased absorption at shorter wavelengths (below 500 nm) due to the absorption of CdS domains. In contrast, Janus NCs synthesized at 200 °C show small blue shifts (<2 nm) in both the absorption and PL spectra (Figure 3b). This can be ascribed to the partial Cd‐doping in the CsPbBr_3_ domain and the broadening of band gap due to lattice contraction, as described in previous reports.^[^ ^40^ ^]^ We also compared the PL lifetimes of Janus NCs and corresponding control CsPbBr_3_ NCs (Figure S17 and Table S2, Supporting Information). In some cases, the Janus and control NCs may be synthesized under different reaction conditions to achieve the same targeted edge lengths of the CsPbBr_3_ domains. This may introduce different surface/defect states and PL decay dynamics. Nonetheless, comparing their PL lifetimes still allows for qualitative understanding of their different decay behaviors due to the formation of heterointerfaces. As shown in Table S2 in the Supporting Information, Janus NCs generally show two‐ to threefold longer averaged PL lifetime than the control samples. This suggests the heterostructures facilitate long‐lived charge separation at the interface, which also increases the percentage of long‐lived decay by using biexponential fit. Prolonged PL lifetime was also observed in type II heterostructures between CsPbBr_3_ and metal chalcogenide NCs^[^ ^26^, ^29^ ^]^ or conventional metal chalcogenide core/shell QDs.^[^ ^41^ ^]^ We also noted that the averaged PL lifetime increased with the sizes of Janus or control NCs. This can be ascribed to the enhanced charge carrier confinement in smaller NCs,^[^ ^42^ ^]^ especially when the sizes of the CsPbBr_3_ domain are in the quantum confinement regime. Additionally, we did not observe Stokes‐shifted emission in the Janus NCs compared to control samples. Although Stokes‐shifted emission peaks are typically associated with conventional type II core/shell NCs based on II–VI semiconductors,^[^ ^41^, ^43^, ^44^ ^]^ they were not observed in recently reported type II or quasi‐type II NC heterostructures between CsPbBr_3_ and CdS^[^ ^26^, ^27^ ^]^ or ZnS.^[^ ^29^ ^]^ Possible origins for this contrast lie in the relatively small differences in the band gaps and band positions between CsPbBr_3_ and CdS or ZnS, together with complexities in size inhomogeneity, Cd‐doping, and others.

Figure 3c depicts the schematic band alignment of CdS (conduction band minimum or CB, about −0.9 eV and valence band maximum or VB, 1.6 eV, vs reversible hydrogen electrode, RHE) and CsPbBr_3_ (CB and VB, −1.0 and 1.4 eV vs RHE) based on reported data^[^ ^45^ ^]^ and DFT calculations (as discussed later). Although the band alignments depend on the sizes of each domains and vary from different literatures, the alignments in Figure 3c are (semi‐)quantitatively valid for showing the relative positions of bands of the two components. This is because the sizes of CsPbBr_3_ and CdS domains in our Janus NCs are mostly in the weakly quantum confined regime. According to this scheme, excitation at 510 nm can produce excitons in the CsPbBr_3_ domains but not CdS. Higher energy photons can simultaneously excite both domains. In either case, the photogenerated electrons are expected to transfer to CdS while holes are localized at the CsPbBr_3_ domains, forming type II heterostructures. This promoted charge separation process at the heterointerface may contribute to the lower PL intensities of Janus NCs relative to plain CsPbBr_3_ NCs. The PL of 200 °C Janus NCs was strongly reduced compared to the control samples (i.e., 16 nm CsPbBr_3_ NCs) synthesized under similar condition.

We conducted TA measurements to probe the electron transfer rate between the coupled CdS and CsPbBr_3_ domains in the type II Janus NCs. Figure 3d–f shows the TA spectra of Janus NCs synthesized at 160 and 200 °C, and 16 nm CsPbBr_3_ NCs, respectively, in the range of ≈1 ps to 6 ns. Small and large Janus NCs were used in the following discussions to denote samples synthesized at 160 and 200 °C, respectively. To simplify our analysis, we used a pump wavelength of 510 nm that only excited the CsPbBr_3_ domains without affecting CdS.^[^ ^46^ ^]^ The TA spectra of plain CsPbBr_3_ NCs (Figure 3f) show an exciton bleach (or ground state bleach) at ≈510 nm, corresponding to the state filling of CB electrons and VB holes.^[^ ^47^ ^]^ An additional bleach at ≈440 nm was observed in the case of Janus NCs synthesized at 160 °C, which forms in a few ps (Figure 3d and Figure S18, Supporting Information). This feature originates from the transfer of photogenerated electrons in CsPbBr_3_ to fill the CB of CdS, corroborating the proposed type II alignment. Large Janus NCs synthesized at 200 °C showed smaller ground state bleach features related to CdS at slightly longer wavelength (≈455 nm, Figure 3f,g), probably due to the much higher extinction coefficients of the CsPbBr_3_ domain than the CdS domain, thus dwarfing the relative amplitude of the CdS bleach feature. Figure 3h shows a comparison of the exciton bleach recovery kinetics in plain CsPbBr_3_ and in small and large Janus NCs. Although in general Janus NCs show faster bleach recovery compared to plain NCs, it is interesting to note that the decay is considerably faster in small Janus NCs. This likely stems from a stronger electronic coupling at the CsPbBr_3_/CdS interface and/or a larger electron transfer driving force for small CsPbBr_3_ NCs. Further studies are necessary to probe the origin of this size‐dependent effect.

An unambiguous assignment of the interdomain electron transfer was provided by an excellent correlation between the kinetics of CsPbBr_3_ bleach decay and CdS bleach formation observed in Janus NCs synthesized at 160 °C (Figure 3i). Considering that the CdS bleach feature at ≈440 nm is initially overlapped with the CsPbBr_3_ photoinduced absorption feature, we performed a subtraction under a reasonable assumption that the photoinduced absorption and exciton bleach features of CsPbBr_3_ share the same kinetics. After subtraction, we obtained a pure kinetics probed for the CdS bleach (blue line in Figure 3i). By fitting this kinetics, we obtained an electron transfer time constant of 8.9 ± 1.4 ps and a slow recombination (>10 ns) of the charge‐separated state. Fitting the kinetics of the CsPbBr_3_ bleach (black line in Figure 3i) reveals a consistent electron transfer time constant of 8.9 ± 0.8 ps. We thus concluded that the interdomain electron transfer takes place in ≈9 ps.

We also collected the TA spectra for Janus NCs at the pump wavelength of 400 nm, which are sufficiently energetic to excite both CsPbBr_3_ and CdS domains. Notable bleach of exciton band in CdS was observed for Janus NCs (Figure S19, Supporting Information). The wavelengths for these CdS bleach features were about 440 and 455 nm, respectively, matching those observed at the pump wavelength of 510 nm. The kinetics for CdS bleach in the large Janus NCs is also slightly slower than that of small Janus NCs. In contrast, the TA spectra of CsPbBr_3_ NCs showed only bleach features related to CsPbBr_3_. These TA results clearly show the co‐existence of CdS and CsPbBr_3_ in Janus NCs and their interdomain charge separation enabled by a type II band alignment.

Temperature‐dependent PL spectra provided additional insights on the exciton kinetics of these Janus NCs. As shown in Figure S20 in the Supporting Information, the PL peaks for 16 nm CsPbBr_3_ NCs blue‐shift (i.e., higher energy) at higher temperatures. This can be explained by the empirical Varshni model; the band gaps of CsPbBr_3_ increase with temperature due to the combination of lattice thermal expansion and the antibonding nature of CB/VB bands.^[^ ^48^ ^]^ The highly asymmetric PL spectra collected at low temperatures (<50 K) can be devolved into two components (Figure S20e, Supporting Information). The one with higher energy corresponds to the band edge emission (P1 for abbreviation) while the other originates from recombination involving shallow surface states (P2).^[^ ^49^ ^]^ These two pathways merge at temperatures over 100 K, primarily due to the thermally assisted activation of excitons. The PL peaks of CsPbBr_3_/CdS Janus NCs synthesized at 150, 160, and 200 °C also blue‐shift with increased temperatures (Figure S20a–c, Supporting Information). However, the PL peaks remain mostly symmetric with a single component at all temperatures. As shown in Tables S3 and S4 in the Supporting Information, P2 dominates the recombination at low temperatures for the Janus NCs. The absence of band edge P1 in Janus NCs may be related to the electron transfer from the CB of CsPbBr_3_ to CdS, as described in Figure S20f in the Supporting Information. Note the origin of the asymmetric PL peaks for perovskites is still under debate. For instance, a recent work assigns them to the changes in the preferential localization of Cs^+^ cations at different temperatures.^[^ ^50^ ^]^ In our experiments, no peak splitting was observed for Janus NCs, which thus made the Cs^+^ localization mechanism less important. Ongoing work is to unambiguously explore the exciton kinetics and pathways. Nonetheless, results from the temperature‐dependent PL spectra support the proposed efficient charge separation in the epitaxial type II Janus NCs.

### 2.3 DFT and NA‐MD Modeling of the Epitaxial Interactions

**Figure 4:**
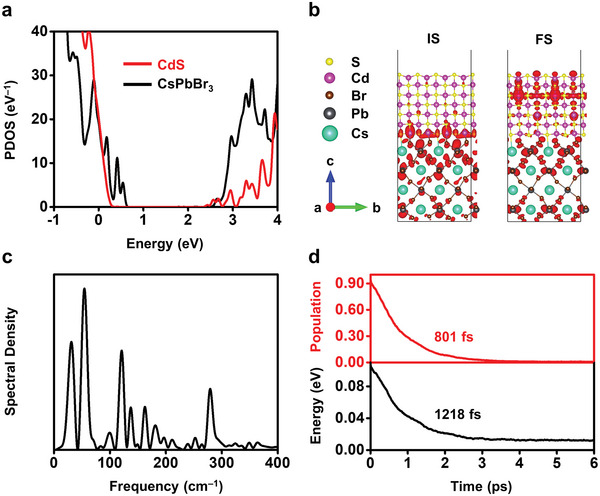
DFT and NA‐MD modeling of the electronic structures and charge transfer dynamics in Janus NCs. a) PDOS of the CsPbBr_3_/CdS heterojunctions, obtained using the optimized geometry shown in Figure S21 in the Supporting Information. The zero energy is set to the Fermi level. b) Charge densities of the IS and FS for the electron transfer. Charge densities are represented by red shapes. c) Spectral density identifies the phonon modes coupled to electronic subsystem, computed as Fourier transforms of the un‐ACF for the phonon‐induced fluctuations of the IS‐FS energy gap. d) Evolution of population (red curve) and energy (black curve) of the IS for the electron transfer. Electron transfer is faster than energy relaxation, which maintains the electrons “hot” over 400 fs for minimizing nonradiative charge losses.

Results from DFT and molecular dynamics (NA‐MD) modeling corroborate the proposed rapid electron transfer at the interface of CsPbBr_3_/CdS Janus NCs. We calculated the projected density of states (PDOS) using the optimized geometry presented in Figure S21 in the Supporting Information. **Figure** **4**a shows that CsPbBr_3_ and CdS form a type II alignment with a small offset (≈0.1 eV) between the CBs of two materials. This offset is in excellent agreement with that measured in previous reports.^[^ ^27^ ^]^ The CBs of CsPbBr_3_ and CdS correspond to the initial state (IS) and final state (FS) for electron transfer across the interface, whose charge densities are shown in Figure 4b. The IS charge density is primarily localized on the Br atoms of the CsPbBr_3_ domain, while the FS state shows significant charge density on the S atoms of the CdS domain. This transition correlates well with the proposed strong coupling at the type II interface and the rapid charge transfer from CsPbBr_3_ to CdS.

Thermal fluctuations further strengthen the interfacial interaction (Figure S21, Supporting Information) and accelerate electron transfer. Figure 4c shows the computed spectral densities that identify the phonon modes participating in the electron transfer across the CsPbBr_3_/CdS interface. Details are shown in the Supporting Information. The spectra are dominated by low‐frequency vibrational modes below 400 cm^−1^. These peaks are identified as, distortion and vibration of [PbBr_6_]^4−^ (33 and 72 cm^−1^),^[^ ^51^, ^52^ ^]^ bending and stretching of Pb—Br bonds (130 and 200 cm^−1^),^[^ ^53^ ^]^ Cs atomic motions (110–130 cm^−1^),^[^ ^51^ ^]^ and vibration in CdS (≈300 cm^−1^).^[^ ^54^, ^55^ ^]^ Beyond these, an evident mode for Cd—Br stretching appears at 365 cm^−1^,^[^ ^56^ ^]^ indicating the strong interfacial interactions between the two domains. These multiple phonon modes produce strong NA coupling in addition to the well‐mixed IS and FS wavefunctions and promote electron transfer.

Figure 4d compares the rates of electron transfer (top panel) and intraband energy relaxation (bottom panel) at the interface. The time constants are obtained by exponential fitting, *P*(*t*) = exp ( − *t*/*τ*) and *E*(*t*) = *E*(0)exp ( − *t*/*τ*), respectively. *P*(*t*) and *E*(*t*) are time‐dependent population and energy of the IS. *E*(0) is the average energy of the CBM of CsPbBr_3_ relative to the CBM of CdS (set as 0 eV). As shown in Figure 4d, electron transfer occurs rapidly at ≈801 fs, faster than the corresponding energy loss (1218 fs). This guarantees the extraction of “hot” electrons from CsPbBr_3_ to CdS before relaxation/cooling. Note that carrier relaxation is one of the primary energy loss mechanism in single junction perovskite solar cells and applications involving photoelectric conversions.^[^ ^21^, ^57^ ^]^ Suppression of this process by efficient charge transfer is critical in improving the performance of relevant devices. The relatively long relaxation time of the “hot” electrons may allow rapid, bandlike transport at long distances, supporting the observed enhanced photoconducting behavior, as discussed below.

### 2.4 Efficient Photoconductors based on Epitaxial Janus NCs

**Figure 5:**
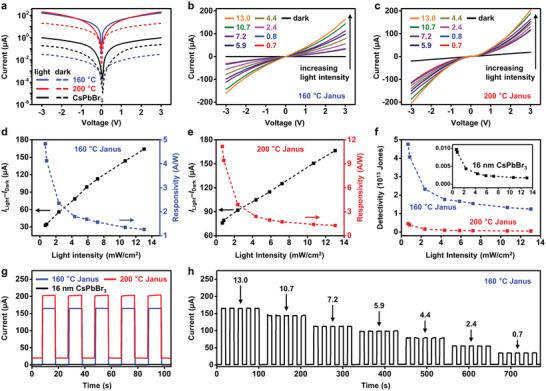
Photoconductors based on Janus NCs. a) *I*–*V* characteristics of thin‐film photoconductors made from Janus NCs (synthesized at 160 and 200 °C) and 16 nm CsPbBr_3_ NCs, under dark (dashed lines) and illumination (450 nm, 13.0 mW cm^−2^, solid lines). The external bias is 3.0 V. b,c) *I*–*V* characteristics under different light intensities (from 0.7 to 13.0 mW cm^−2^) for devices based on Janus NCs. d–f) Photocurrent, responsivity, and detectivity of devices based on Janus NCs synthesized at 160 and 200 °C, respectively. Inset in (f) shows the detectivity of devices based on 16 nm CsPbBr_3_ NCs. g) On–off switching behavior of devices operated under the bias of 3.0 V and illumination light intensity of 13.0 mW cm^−2^. h) On–off switching of devices based on Janus NCs synthesized at 160 °C under varied light intensities.

The type II epitaxial heterostructures promote the spatial decoupling of photogenerated electrons and holes in Janus NCs. This process favors photoelectrical conversion and photochemical transformation, with broad implications in photodetection and photocatalysis. As a proof of concept, we fabricated photoconductor devices by spin‐coating CsPbBr_3_ and Janus NC thin films on silicon substrates with predesigned Au interdigitated electrodes (10 µm wide, 100 µm long). Devices based on Janus NCs show substantially increased photocurrents (two to three orders of magnitude) compared to those with CsPbBr_3_ NCs (**Figure** **5**a and Figure S22, Supporting Information). Figure 5b,c shows the current–voltage (*I*–*V*) characteristics of Janus NC films (synthesized at 160 and 200 °C) under various light intensities (from 0.7 to 13.0 mW cm^−2^). Higher light intensities corresponded to larger photocurrents. Responsivity (*R*) and detectivity (D*) represent the capabilities of a photodetector to respond to light, and the lowest level of light that can be detected, respectively. *R* was calculated as the ratio of photocurrent to incident light intensity

(1) $$R=I_{\mathrm{ph}}/PS$$

where *I*_ph_ is the photocurrent that can be calculated from the difference in *I*_light_ and *I*_dark_, *P* is the light intensity, and *S* is the effective illuminated area. Due to the limitation of our instruments, D* was calculated based on *I*_dark_ by using the formula

(2) $$D*=R/{\left(2qI_{\mathrm{dark}}\right)}^{1/2}$$

where *q* is the elementary charge. Note the measured D* by using *I*_dark_ can be two to three orders of magnitude higher (i.e., overestimated) than those measured by using Flicker noise.^[^ ^58^ ^]^ Nonetheless, the comparison of D* of CsPbBr_3_ and Janus NC‐based devices, both were measured by using *I*_dark_, can still provide important information on the effect of NC heterointerface on device detectivity. Figure 5d–f and Table S5 in the Supporting Information compare these parameters for CsPbBr_3_ and Janus NC‐based devices. Devices based on Janus NCs synthesized at 160 °C show high *R* (up to ≈4.8 A W^−1^) and D* (4 × 10^13^ Jones), which are approximately three orders of magnitude higher than CsPbBr_3_ NC‐based devices. These values, achieved with a simple device configuration, are comparable with the state‐of‐the‐art, CsPbBr_3_ (NC or other forms) based photoconductors with additional charge transport layers/functional materials (Table S6, Supporting Information). Note D* listed in Table S6 in the Supporting Information was also calculated by using *I*_dark_, except in one report. Devices based on Janus NCs synthesized at 200 °C demonstrated a higher responsivity *R* (≈11.2 A W^−1^ measured at 0.7 mW cm^−2^) but lower D*, compared to devices made from Janus NCs synthesized at 160 °C. This is due to their higher *I*_dark_, probably related to defect states of the 200 °C synthesized Janus NCs. Figure 5g shows the current–time (*I*–*t*) curves under 450 nm light illumination (13.0 mW cm^−2^) at a bias of 3.0 V. *I*_light_ of 160 and 200 °C synthesized Janus NCs is ≈270 times higher than that of 16 nm CsPbBr_3_ NCs. We calculated the external quantum efficiency (EQE), defined as the ratio of the number of charge carriers collected by the photodetector at a fixed bias to the number of photons of a given light beam, for these devices.

(3) EQE=Rhυ/e

In Equation (3), *hυ* is the energy of the incident photons and *e* is the absolute value of the electron charge. Figure S23a in the Supporting Information shows the EQEs of devices under different light intensities for CsPbBr_3_ and Janus NC‐based devices. Janus NC‐based devices show two to three orders of magnitude higher EQEs that those based on CsPbBr_3_ NCs. These devices also show fast and stable photoresponses at different light intensities without hysteresis. The response speed (rise and decay times) for device based on 160 °C Janus NCs with a bias of 3.0 V and light intensity of 13.0 mW cm^−2^ was measured by using the smallest time interval for data collection (Δ*T* = 1.6–2 ms) in our instrument. The measured rise/decay response time of 1.6 ms (Figure S23b, Supporting Information) approaches the measurement limit of the instruments. The actual response time should be shorter than 1.6 ms. The devices can operate continuously for at least 30 min, as shown by the on–off current cycles under continuous irradiation at various or constant light intensities, in ambient atmosphere (Figure 5h and Figure S24a,b, Supporting Information). They also show decent storage stability. After stored under ambient atmosphere (room temperature of ≈20 °C and humidity of ≈35%) for 7 days, devices maintained >90% of their initial photocurrent (Figure S24c,d, Supporting Information). The above data show the potential of translating the fast charge separation in epitaxial Janus CsPbBr_3_/CdS NCs to improved performance in the photoconductor device model and other optoelectronic devices. As shown in other NC‐based devices,^[^ ^18^ ^]^ the device performance relies on not only the intrinsic properties of NCs but also the film qualities, interfacial behaviors, and others. Further device‐oriented studies are necessary for better device performance and clear connections between device performance, materials properties, and synthetic parameters. For instance, device optimizations such as introducing additional transport layers are expected to further enhance the performance of Janus NC‐based devices.^[^ ^59^ ^]^

### 2.5 Synthetic Versatility of Janus NCs

The synthesis of Janus NCs can be extended to CsPbX_3_/CdS (X = Cl, I) and CsPbBr_3_/ZnS. On the one hand, post‐synthetic treatment by exposing Janus NCs to Cl^−^ and I^−^ anions^[^ ^60^, ^61^ ^]^ can readily transform the CsPbBr_3_ domain to corresponding metal halide perovskites. The anion exchange was evidenced by the color change and shifts in the UV‐visible absorption spectra (Figure S25, Supporting Information). Impressively, the chemical transformation does not alter the structures and/or impair the epitaxial interfaces. This may arise from the “lattice anchoring” effect in epitaxial heterostructures, as revealed in the increased structural and phase stability of perovskites in heterostructures, especially the metastable *α*‐CsPbI_3_.^[^ ^34^, ^62^ ^]^ On the other hand, CsPbBr_3_/ZnS Janus NCs can be synthesized following similar procedures but by replacing Cd(OA)_2_ for Zn(OA)_2_ (Figure S26, Supporting Information). The shape of CsPbBr_3_/ZnS resembles that of CsPbBr_3_/CdS and the typical sizes for CsPbBr_3_ and ZnS domains are ≈6 and ≈9 nm, respectively. Despite the lattice mismatch between ZnS and CsPbBr_3_ (≈4.2%), we expect to obtain high‐quality CsPbBr_3_/ZnS Janus NCs and probably those with ternary components (CsPbBr_3_/CdS/ZnS) after optimization.

## 3 Conclusion

In conclusion, we report the synthesis of epitaxial CsPbBr_3_/CdS Janus NCs with a type II band alignment, tailored for ultrafast charge separation and extraction at the interface. The uniform NC heterostructures show evident epitaxial relation, precisely adjustable sizes, and readily tunable optical properties, which are not available in their counterparts in previous reports. Following from the rapid charge separation (≈9 ps) at the strongly coupled heterointerfaces, Janus NC‐based devices show significantly improved photoconducting behavior (approximately three orders of magnitude increase in *R* and D*). The compositions of epitaxial heterostructures can be extended to other metal halide perovskites and metal chalcogenides, leading to potentially different band alignments and versatile wavefunction engineering that are hard to achieve in solely perovskite NCs. Results in this work complement the remarkable progress on perovskite (only) NCs and open up a new designer space for perovskite NC materials toward advanced functionalities in optoelectronics, photocatalysis, and related fields.

## Conflict of Interest

The authors declare no conflict of interest.

## Supporting information

Supporting Information

## Data Availability

The data that support the findings of this study are available from the corresponding author upon reasonable request.
