# Supplementary material for: Epitaxial CsPbBr3/CdS Janus Nanocrystal Heterostructures for Efficient Charge Separation
Source: Adv Sci (Weinh). 2023 Feb 25;10(13):2206560. doi: 10.1002/advs.202206560 (PMC10161108; doi:10.1002/advs.202206560)
Supplement: Supplementary file 1 — Supporting Information [file ADVS-10-2206560-s001.pdf]

Supporting Information

Epitaxial $CsPbBr_3$/CdS Janus Nanocrystal Heterostructures for Efficient Charge Separation

Hengwei Qiu, Fu Li, Shan He, Ran Shi, Yaoyao Han, Hannikezi Abudukeremu, Lin Zhang, Yan Zhang, Song Wang, Wangyu Liu, Chao Ma, Honghua Fang, Run Long, Kaifeng Wu, Hao Zhang,* Jinghong Li*

Dr. H. Qiu, Dr. F. Li, H. Abudukeremu, Dr. S. Wang, W. Liu, Dr. C. Ma, Prof. H. Zhang, Prof. J. Li
Department of Chemistry, Center for BioAnalytical Chemistry, Key Laboratory of Bioorganic Phosphorus Chemistry & Chemical Biology of Ministry of Education, Tsinghua University, Beijing 100084, China
E-mail: hzhangchem@mail.tsinghua.edu.cn

S. He, Y. Han, Prof. K. Wu
State Key Laboratory of Molecular Reaction Dynamics, Dalian Institute of Chemical Physics, Chinese Academy of Sciences, Dalian 116023, China

S. He, Y. Han, Prof. K. Wu
University of the Chinese Academy of Sciences, Beijing 100049, China

R. Shi, Dr. L. Zhang, Prof. R. Long
College of Chemistry, Key Laboratory of Theoretical & Computational Photochemistry of Ministry of Education, Beijing Normal University, Beijing 100875, China
E-mail: runlong@bnu.edu.cn

Y. Zhang, Prof. H. Fang
Department of Precision Instrument, State Key Laboratory of Precision Measurement Technology & Instruments, Tsinghua University. Beijing 100084, China

Experimental Section

Chemicals. Cesium carbonate (Cs_2CO_3, 99.9%, trace metals basis), lead(II) chloride ($PbCl_2$, 99.999%, trace metals basis), lead(II) bromide ($PbBr_2$, 99.999%, trace metals basis), lead(II) iodide (PbI_2, 99.999%, trace metals basis), cadmium oxide (CdO, 99.99%, trace metals basis), sulfur powder (S, 99.998%, trace metals basis), hexane (anhydrous, 95%), methyl acetate (anhydrous, 99.5%), and N,N-dimethylformamide (DMF, anhydrous, 99.8%) were purchased from Sigma-Aldrich. Oleic acid (OA, tech. 90%), 1-octadecene (ODE, tech. 90%), and 1-dodecanethiol (DDT, 98%) were purchased from Alfa Aesar. Oleylamine (OLAM, C18-content 80–90%) was purchased from Acros. Trioctylphosphine (TOP, 97%) was purchased from Strem. All chemicals were used as received without further purification.

Synthesis of molecular precursors. The synthesis of molecular precursors followed reported protocols.[1-3] For the synthesis of Cs-oleate, Cs_2CO_3 (652 mg, 2 mmol), OA (2.5 mL), and ODE (17.5 mL) were added into a 100 mL three-neck flask and dried under vacuum at 110 °C for 1 h. The mixed solution was then heated at 150 °C under N_2 until it turned clear. The formed Cs-oleate solution was then transferred into glovebox and kept under inert atmosphere before further use.

$Cd(OA)_2$ was synthesized by dissolving CdO (383 mg, 3 mmol) in a mixture of ODE (3.9 mL) and OA (3.9 mL) in a 25 mL three-necked flask. The mixture was degassed at 100 °C for 30 min before heated to 280 °C under N_2 atmosphere. The formed clear solution was degassed at 120 °C for 30 min, yielding a white, waxy solid of $Cd(OA)_2$ at room temperature. $Zn(OA)_2$ precursor was synthesized by a similar procedure by reacting $Zn(Ac)_2{\cdot}2H_2O$ (439 mg, 2 mmol) with OA (1.3 mL) in ODE (18.7 mL). S-ODE was made by dissolving S powder (48 mg, 1.5 mmol) in dried ODE (15 mL) in a glass vial under N_2.

$PbBr_2$ solution for synthesizing $CsPbBr_3$ nanoclusters was prepared by dissolving $PbBr_2$ (734 mg, 2 mmol) in a mixture of OA (5 mL), OLAM (5 mL) and ODE (30 mL) in a 100 mL three-necked flask. The mixture was dried under vacuum at 110 °C for 30 min, followed by heated to 150 °C under N_2 until fully dissolved. The formed solution was cooled to room temperature and kept in glovebox. Solutions of $PbCl_2$ and PbI_2 used in anion exchange were prepared by dissolving corresponding lead salts in ODE with OA and OLAM ligands at elevated temperatures (up to ~150 °C). In the case of $PbCl_2$, TOP was added to assist the solubilization.

Synthesis of $CsPbBr_3$ nanoclusters. The synthesis of $CsPbBr_3$ nanoclusters used the method reported by Pradhan and co-workers.[2] The Cs-oleate solution (0.2 mL) was injected

to the $PbBr_2$ solution (4 mL), followed by stirring at room temperature. The absorption features of the solution was monitored during the reaction. The reaction was quenched when the product showed an absorption peak at ~400 nm. The formed $CsPbBr_3$ nanoclusters were then centrifuged at 8000 rpm for 5 min and collected as the precipitates. Nanoclusters were redispersed in 1 mL of ODE.

Synthesis of epitaxial $CsPbBr_3$/CdS Janus NCs. A 25 mL three-necked flask containing a solution of $Cd(OA)_2$ (26 μL) and DDT (25 μL) in dried ODE (4 mL) was preheated under N_2 at 150–220 °C (150, 160, 170, 180, 200 and 220 °C), followed by the swift injection of a solution of $CsPbBr_3$ nanoclusters (1 mL) and S-ODE (0.1 mL, 0.1 mM). After a specific reaction time (e.g., 5 min), the reaction was quenched by using ice-water. For syntheses performed at 150, 160 and 170 °C, the crude solution was added with methyl acetate (15 mL) and centrifuged at 10000 rpm for 15 min. The supernatant was discarded and the NC precipitates were redispersed in hexane. For syntheses performed at 180, 200 and 220 °C, the crude solution was centrifuged at 10000 rpm for 5 min and the precipitates were redispersed in hexane. Janus NCs were then purified with an additional wash by using hexane/methyl acetate (1:3 in volume ratio) as solvent/nonsolvent. The purified Janus NCs were redispersed in hexane (5 mL) and left undisturbed for 24 h. The supernatant of these solutions was collected for further characterizations and device fabrication. Reaction parameters, such as the reaction temperatures and time, were adjusted for studying the growth process of Janus NCs.

$CsPbX_3$/CdS (X = Cl, I) NCs via anion exchange. PbX_2 solution ($PbCl_2$ or PbI_2, 150 μL) was added to the $CsPbBr_3$/CdS Janus NC colloids (2 mL). After stirred overnight at room temperature, $CsPbX_3$/CdS (X = Cl, I) NCs were collected by centrifugation at 10000 rpm for 5 min and redispersed in hexane.

Synthesis of $CsPbBr_3$/ZnS NCs. $CsPbBr_3$/ZnS NCs were synthesized following the same procedures for $CsPbBr_3$/CdS NCs. 0.1 mL (0.01 mmol) of $Zn(OA)_2$ was used and the reaction temperature was 220 °C.

TA measurements. Setup for the femtosecond pump-probe TA measurements was described in previous studies.[4] Briefly, the laser source is a regenerative amplified Ti:sapphire laser system (Coherent; 800 nm, 70 fs, 6 mJ/pulse, 1 kHz repetition rate). The 800 nm output pulse was split in two parts with a 50% beam splitter. One part was used to pump an OPA which

generated a wavelength-tunable laser pulse from 250 nm to 2.5 μm as pump beam. Another part was attenuated with a neutral density filter and focused into a sapphire or CaF_2 crystal to generate a white light continuum used for probe beam. The delay between the pump and probe pulses was controlled by a motorized delay stage. The pump pulses were chopped by a synchronized chopper at 500 Hz and the absorbance change was calculated with two adjacent probe pulses (pump-blocked and pump-unblocked). Solutions of Janus NCs and $CsPbBr_3$ NCs were placed in 1 mm cuvettes and measured under ambient conditions.

Temperature-dependent PL measurements. Films of Janus NCs and $CsPbBr_3$ NCs were installed in the optical cryostat (Montana Cryostation s50) and cooled to 12 K with liquid helium. The 400 nm laser with 80 MHz repetition and ~100 fs pulse was used as excitation light source and a spectrometer with CCD (Andor Shamrock 500i) was used to record the PL spectra. The excitation power for every sample was set almost the same.

DFT and NA-MD simulations. The $CsPbBr_3$/CdS NC heterojunction was constructed by interfacing an 88-atom CsBr-terminated $CsPbBr_3$ (3×2) (110) surface with a 112-atom CdS (3×3) (220) surface. Each surface was created using the optimized individual unit cell associated with lattice constant of 5.898 Å for cubic $CsPbBr_3$ (space group *Pm3m*) and 5.810 Å for cubic CdS (space group *Pnma*), which agrees with the experimental value of 5.870 Å and 5.820 Å, respectively.[5-6] This setting leads to minute lattice mismatch of 1.80% and 4.16% along the a- and b-axis, respectively. A vacuum region of 20 Å was added along the heterojunction normal, to avoid spurious interactions between the periodic images.

The *ab initio* nonadiabatic (NA) molecular dynamics (MD) simulation was performed with the fewest switching surface hopping algorithm[7-9] implemented within the framework of time-dependent Kohn-Sham (KS) density functional theory.[10-11] The heavier and slower nuclei were handled semi-classically, while the lighter and faster electrons were described by quantum mechanics.[10–12-13]

The geometry optimization, adiabatic MD, and NA coupling calculations were performed with the Vienna *ab initio* Simulation Package (VASP).[14] The interactions of electron exchange-correlation and electron-ion core were described with the Perdew-Burke-Ernzerhof functional[15] and projector-augmented wave method,[16] respectively. The plane wave basis energy cutoff was set to 400 eV. The Γ-centered 2×3×1 and 4×6×1 Monkhorst-Pack k-point mesh were used for geometry optimization and electronic structure calculations, respectively.[17] The van der Waals interactions are described with Grimme's DFT-D3

approach.[18-19] After geometry optimization, the $CsPbBr_3$/CdS Janus NC was heated to 300 K by repeated velocity rescaling for 2 ps. Then, 6 ps microcanonical ensemble trajectories were generated with a 1 fs atomic time step. The last 2 ps trajectories were chosen for NA coupling calculations and as the initial conditions for the NA-MD simulations.

The spectral densities of thermal fluctuation were computed by performing Fourier transforms of unnormalized autocorrelation function (un-ACF) for the energy gap fluctuations between the IS and FS. The un-ACF is expressed as:

$$C_{ij}(t) = \langle \delta E_{ij}(t') \delta E_{ij}(t - t') \rangle_{t'} \qquad \text{(Equation 1)}$$

where the brackets denote canonical averaging. $\delta E_{ij}(t)$ represents the fluctuation of energy gap between states i and j from the average value. The Fourier transforms are computed as:

$$I(\omega) = \left| \frac{1}{\sqrt{2\pi}} \int_{-\infty}^{\infty} dt\, e^{-i\omega t} C(t) \right|^2 \qquad \text{(Equation 2)}$$

Fabrication and measurements of photoconductors. The substrates for photoconducting devices contained interdigitated Au electrodes with an interval distance of 10 μm. Solutions (15 mg/mL) of $CsPbBr_3$/CdS Janus NCs (synthesized at 160 and 200 °C) or $CsPbBr_3$ NCs (~16 nm) were spin-coated at 1000 rpm for 5 s and 3000 rpm for 5 s. The spin-coating process was performed 3 times to increase the film thickness. Device performance was evaluated by using a Keithley 2400 sourcemeter. A laser diode (450 nm) was employed as the illumination source with tunable light intensities. *I*–*V* curves were measured from –3.0 to 3.0 V. R and D^* of the fabricated photoconductors were extracted from these measurements. The device response time was measured by using a Keysight B1500A semiconductor analyzer. The shortest time interval in data collection in our instrument was limited to ~1.6 ms.

Materials Characterizations. TEM images of NCs were taken using a JEOL JEM-2100F microscope at 200 kV. HAADF-STEM images were collected on a Titan Cubed Themis G2 300 double aberration-corrected TEM microscope at 300 kV. XRD patterns of NC thin films were collected by using a Rigaku Smartlab diffractometer with Cu Kα radiation. Steady-state UV-visible absorption spectra of NC solutions were obtained with an Agilent Cary 5000 UV-Vis-NIR spectrophotometer. Room temperature PL measurements of NC solutions were carried out on a Horiba FluoroMax Plus spectrometer. ICP–OES analysis of digested solutions of NCs was performed on a Varian Vista-MPX spectrometer. The Janus NCs were firstly treated with polar solvents (e.g., N,N-dimethylformamide) to dissolve the $CsPbBr_3$ domains. The CdS hemispheres were then digested by nitric acid.

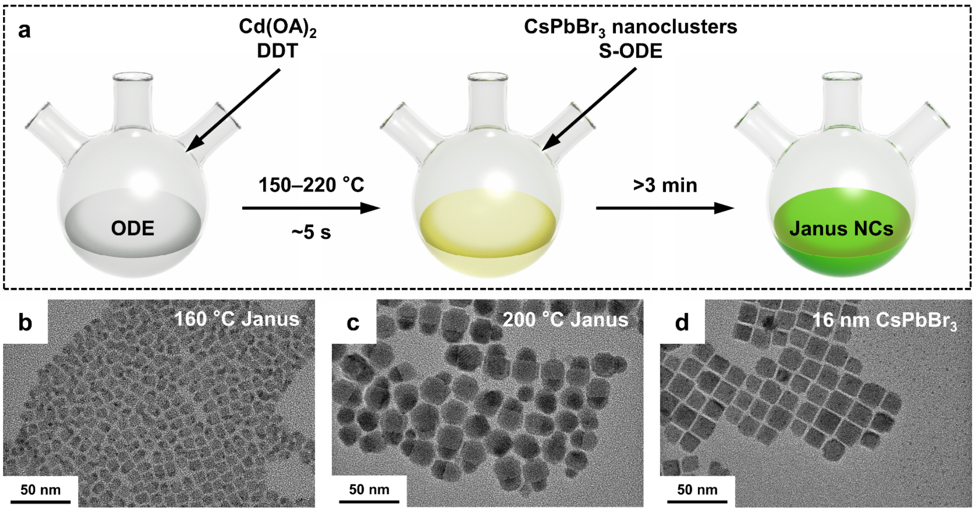

Figure S1. Schematic synthetic procedures and additional TEM images. (a) Scheme of the synthesis procedures. (b–d) TEM images of Janus NCs synthesized at 160 and 200 °C, respectively, and the control sample of 16 nm $CsPbBr_3$ NCs synthesized under similar condition in the absence of $Cd(OA)_2$ and S-ODE.

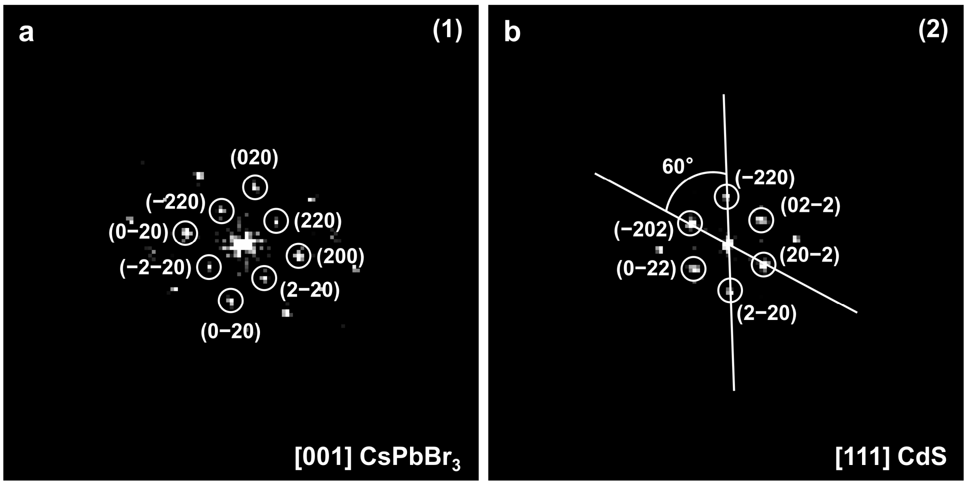

Figure S2. FFT analysis of an individual Janus NC. Detailed analysis of the Janus NC shown in Figure 1c with (a) $CsPbBr_3$ in [001] and (b) CdS in [111] axes. The orientation relation suggests the epitaxial growth of CdS {211} on the {220} facets of $CsPbBr_3$.

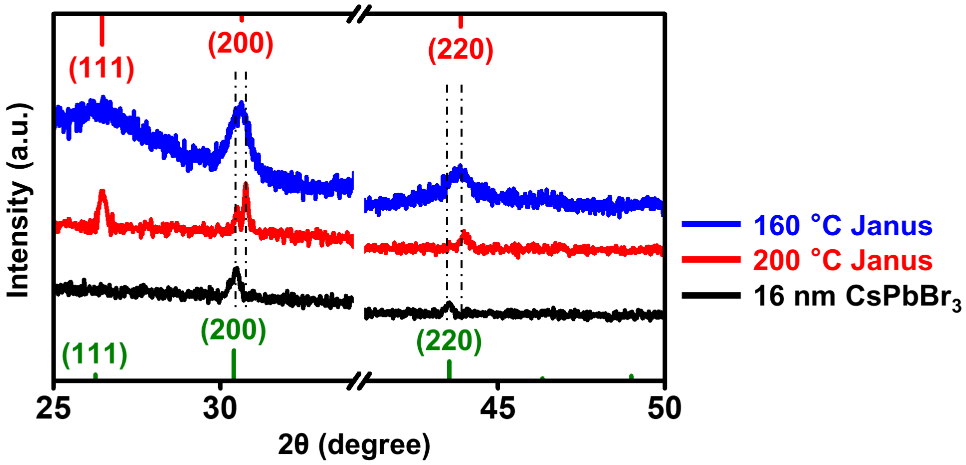

Figure S3. Magnified view of XRD patterns of Janus and $CsPbBr_3$ NCs. Compared to pure $CsPbBr_3$ NCs, new peaks or shifts corresponding to (111), (200), and (220) planes of CdS were observed in Janus NCs. The vertical lines are peaks for $CsPbBr_3$ (bottom, green) and CdS (top, red) standards.

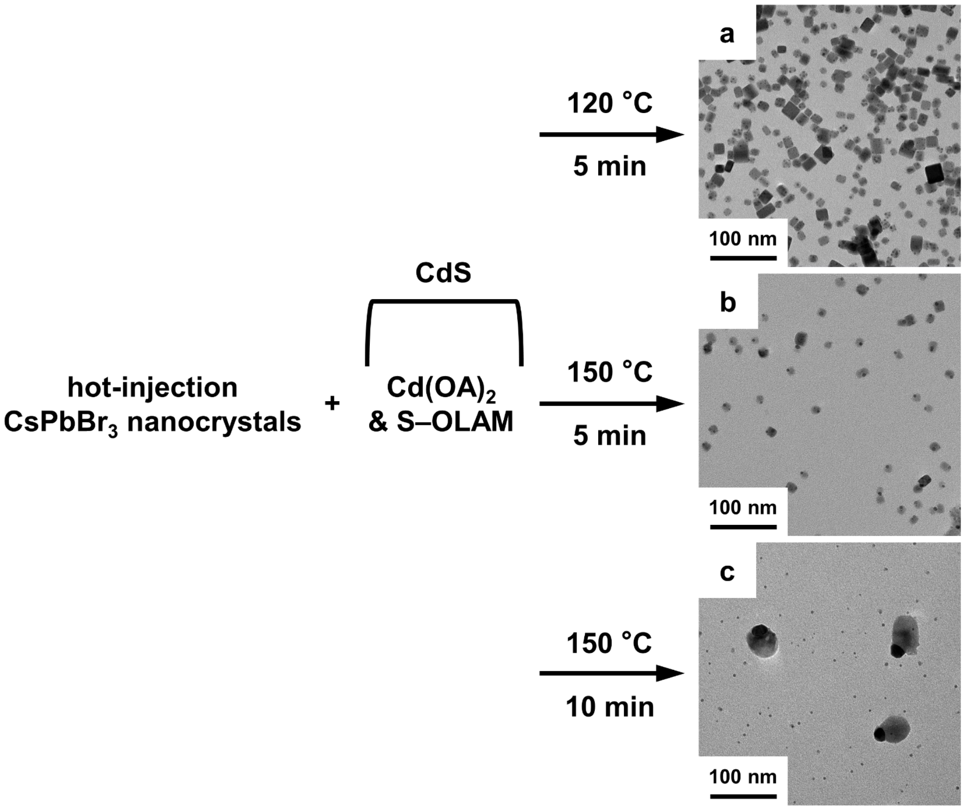

Figure S4. Attempts in the synthesis of Janus NCs with preformed $CsPbBr_3$ NCs as seeds. In brief, the precursors of CdS ($Cd(OA)_2$ and S–OLAM) were added to a flask containing preformed $CsPbBr_3$ NCs obtained via conventional hot-injection method. No or random Janus heterostructures with poor size/shape control were obtained.

The synthesis of Cs-oleate and $CsPbBr_3$ NCs followed reported protocols.[1] For the synthesis of $CsPbBr_3$/CdS Janus NCs, $CsPbBr_3$ NCs (15 mg) were dispersed in dried ODE (4 mL) in a 25 mL three-necked flask. The reaction mixture was preheated under N_2 at 150 or 120 °C, followed by the injection of $Cd(OA)_2$ (26 μL) and S-OLAM (10 μL, 1 M). After 5 or 10 min, the reaction was quenched by using an ice-water bath. The crude solution was centrifuged at 10000 rpm for 5 min and the precipitates were redispersed in hexane followed by purification with hexane/methyl acetate to remove excess ligands. NCs were then used for TEM imaging.

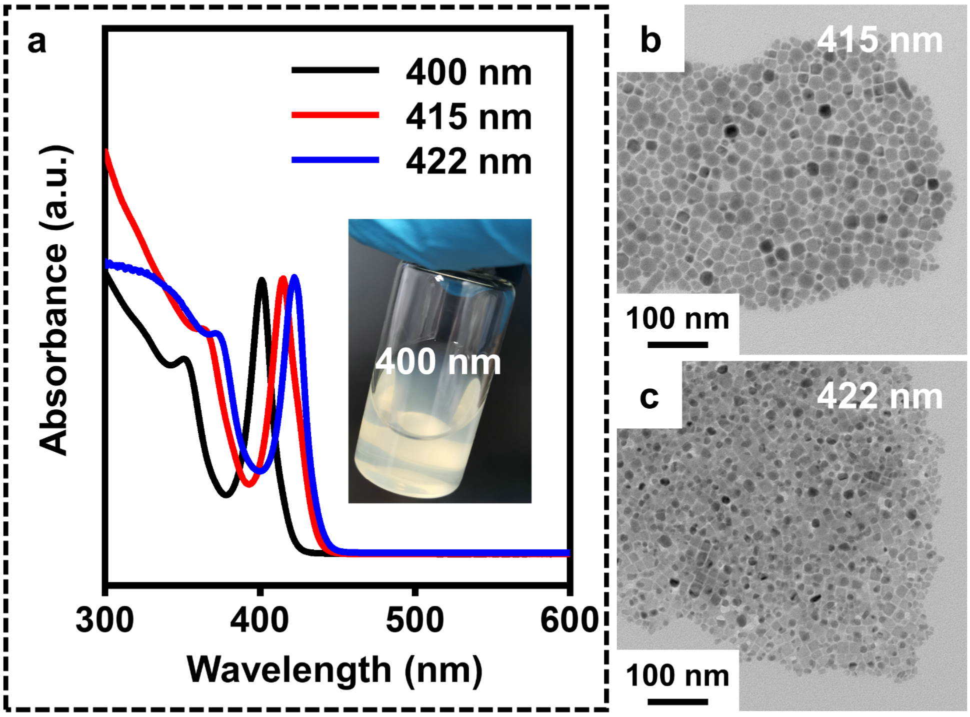

Figure S5. The importance of sizes of $CsPbBr_3$ nanoclusters. (a) UV-visible absorption spectra of nanoclusters with different peak positions (400, 415, and 422 nm), related to their sizes. The inset shows a photograph of the colorless nanocluster solution with the absorption peak at 400 nm. (b,c) TEM images of Janus NCs synthesized with larger $CsPbBr_3$ nanoclusters.

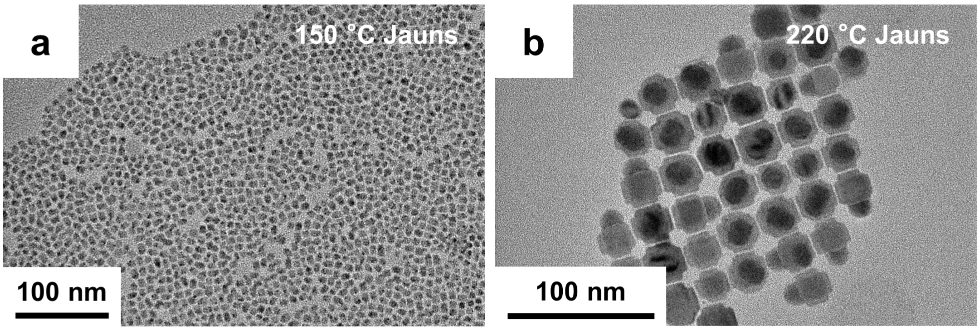

Figure S6. Additional TEM images of Janus NCs synthesized at 150 and 220 °C. The hexapod structures in (b) were from the shape evolution of $CsPbBr_3$ domains at high temperatures, as described in the manuscript.

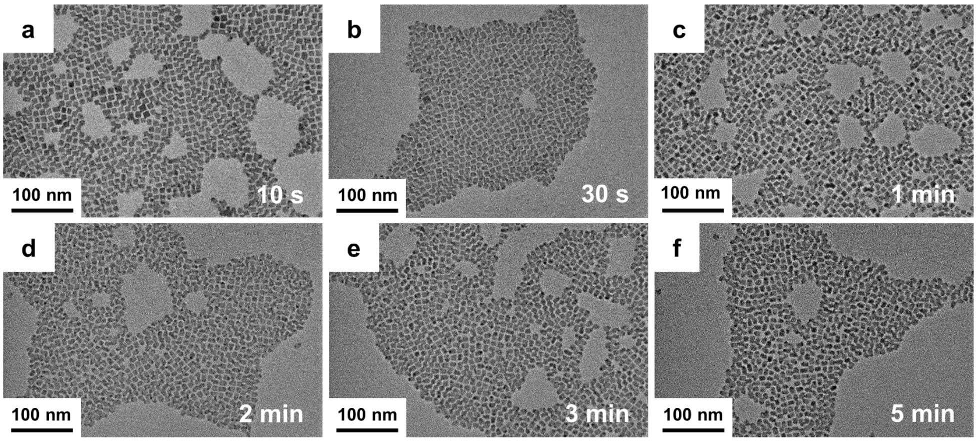

Figure S7. Effect of reaction time on the synthesis of Janus NCs. TEM images of samples synthesized at 160 °C at different reaction time, ranging from 10 s to 5 min.

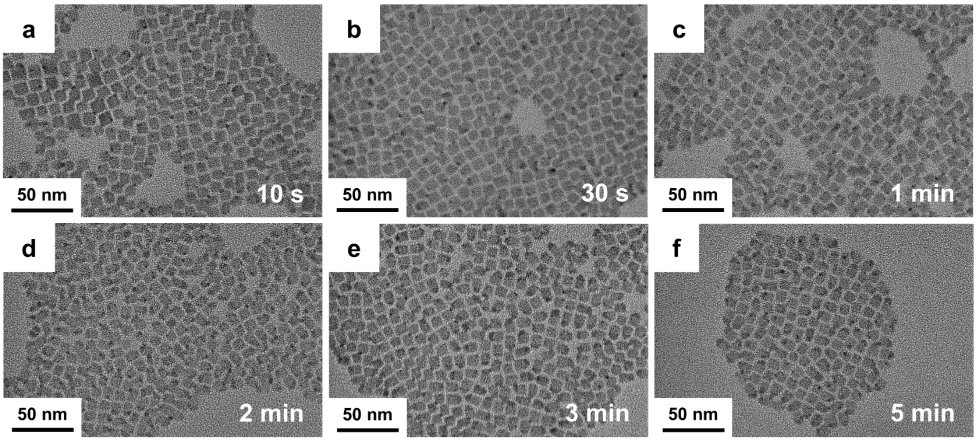

Figure S8. Additional TEM images of Janus NCs synthesized at 160 °C for different reaction time, in higher magnification.

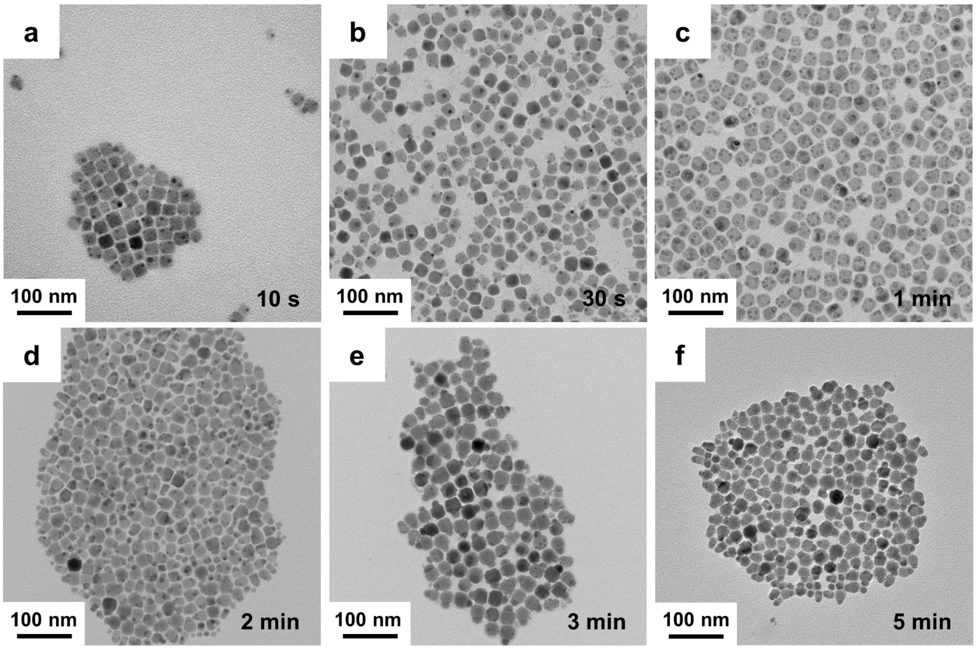

Figure S9. TEM images of Janus NCs synthesized at 200 °C for different reaction time.

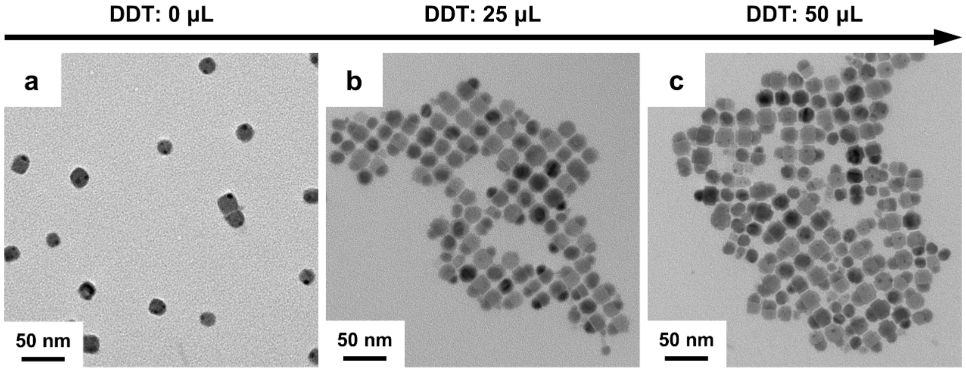

Figure S10. The role of DDT in the synthesis of Janus NCs. TEM images of Janus NCs synthesized at 200 °C for 5 min, with the addition of (a) 0, (b) 25, and (c) 50 μL of DDT.

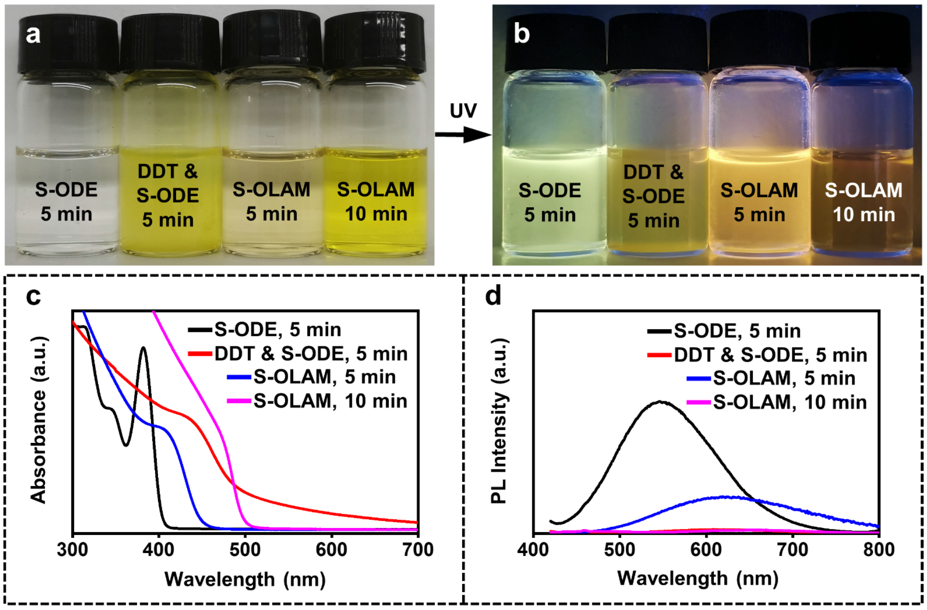

Figure S11. The role of S precursors in the formation of CdS. The choices of S precursors strongly affected the formation kinetics and sizes of CdS. To a solution of $Cd(OA)_2$, different S sources were added and reacted at 200 °C for 5 or 10 min. (a,b) Photographs of the obtained nanoscale CdS samples under ambient and UV light. (c,d) UV-visible absorption and PL spectra of these CdS samples. S-OLAM is the mixture of S in oleylamine.

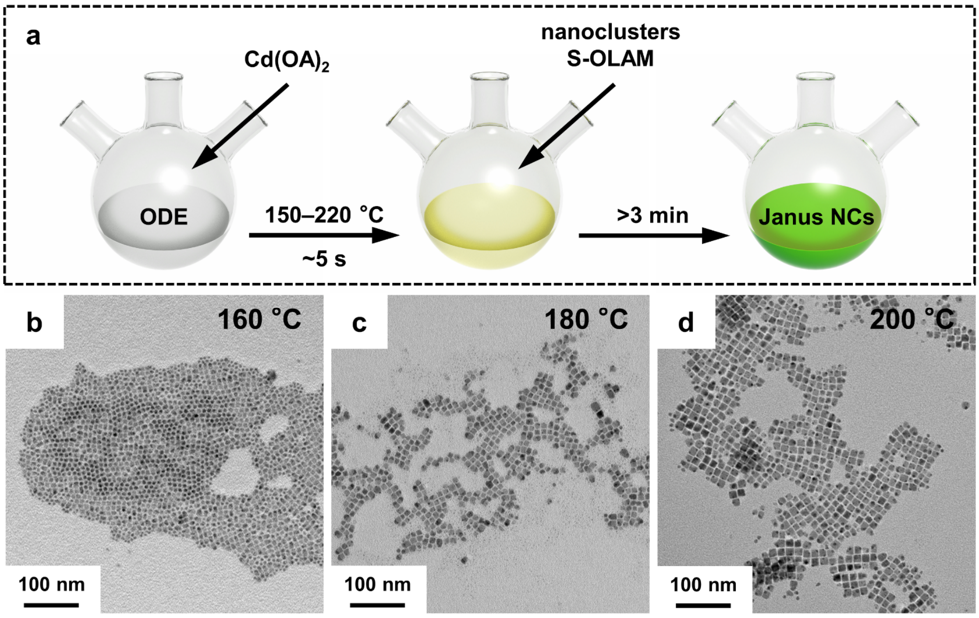

Figure S12. The synthesis of Janus NCs with S-OLAM. (a) Scheme of the synthesis procedures. (b–d) TEM images of Janus NCs synthesized at 160, 180 and 200 °C, respectively, for 5 min. The obtained Janus NCs show much smaller CdS domains compared to those synthesized with S-ODE/DDT. Janus NCs synthesized with S-OLAM precursors also showed inferior size- and shape uniformity compared to those by using S-ODE precursors. Correlating their structures to optical properties requires further optimization in the synthesis.

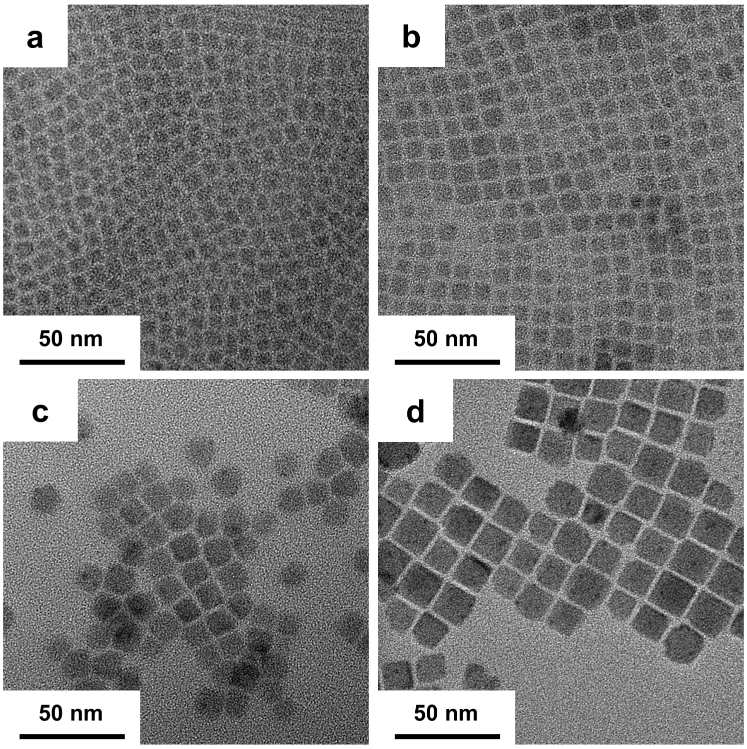

Figure S13. TEM images for control samples of $CsPbBr_3$ NCs with different sizes. (a) 5.5 nm $CsPbBr_3$ NCs, as control to Janus NCs synthesized at 160 °C; (b) 7.6 nm $CsPbBr_3$ NCs, as control to Janus NCs synthesized at 170 °C; (c) 11.1 nm $CsPbBr_3$ NCs, as control to Janus NCs synthesized at 180 °C; (d) 16.1 nm $CsPbBr_3$ NCs, as control to Janus NCs synthesized at 200 °C.

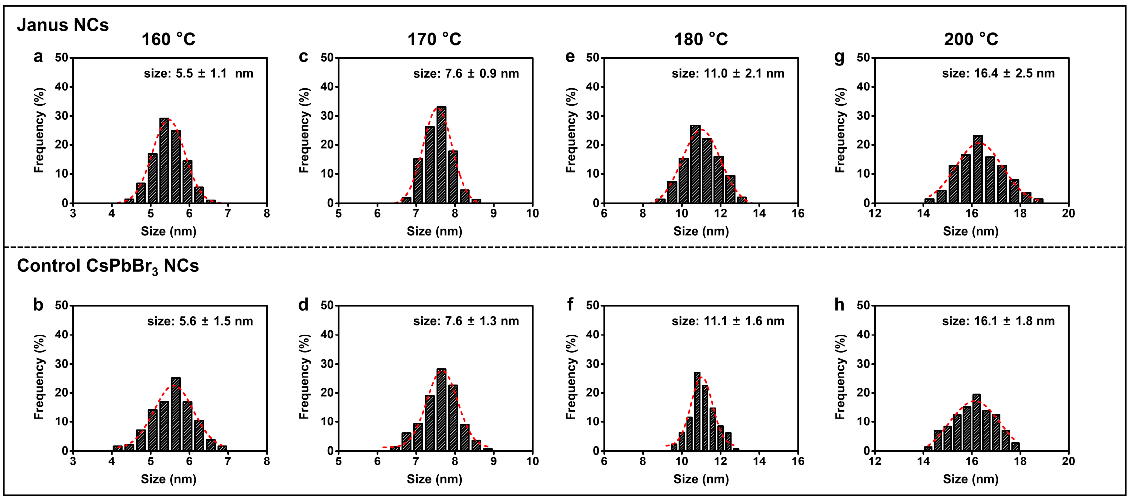

Figure S14. Size distribution analysis of Janus NCs synthesized at different temperatures and corresponding control $CsPbBr_3$ NCs. (Top) Janus NCs and (bottom) the corresponding control $CsPbBr_3$ NCs.

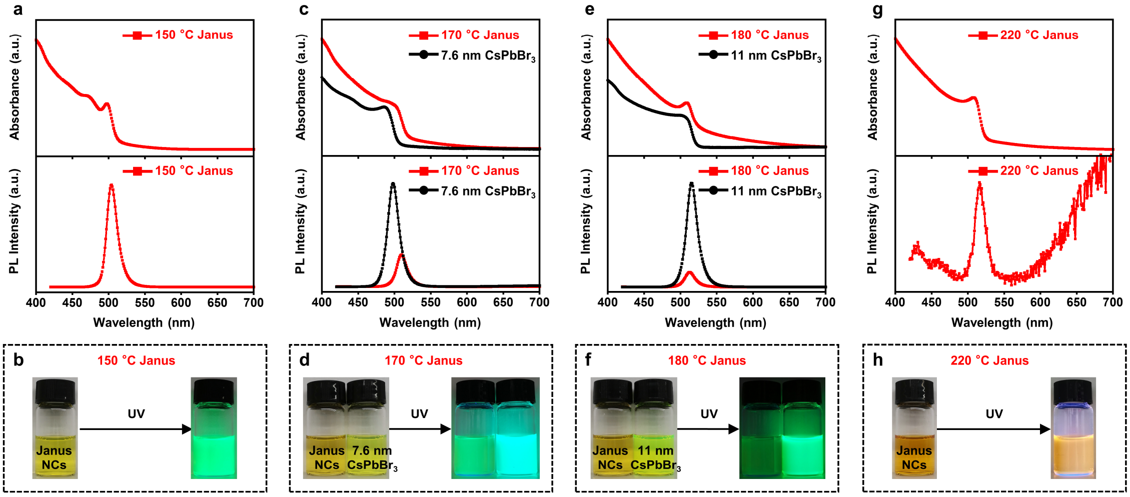

Figure S15. Steady-state optical spectra of Janus NCs and corresponding control samples. (a,c,e,g) UV-visible absorption (top) and PL (bottom) spectra. (b,d,f,h) Photographs of Janus NCs and control samples under ambient and UV light. In (g), the broad emission beyond 600 nm was from the trap states emission of CdS. We confirmed this by treating the Janus NCs with DMF to dissolve $CsPbBr_3$ domains. The obtained CdS NCs also show a broad emission peak in this region.

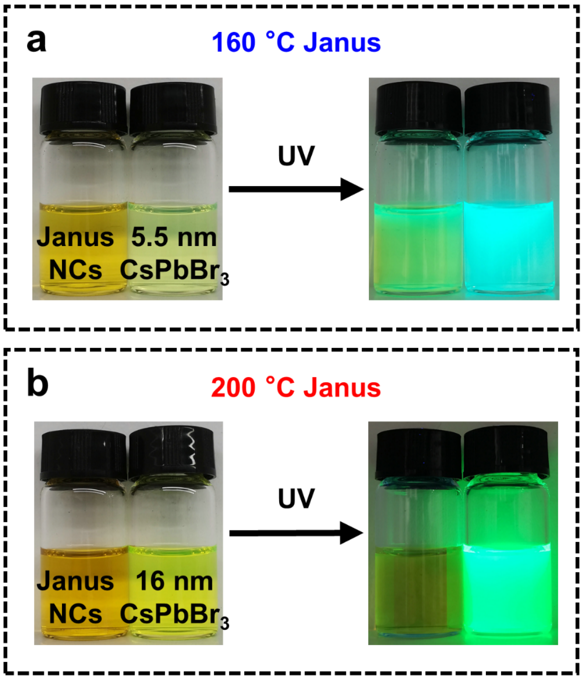

Figure S16. Photographs of solutions of Janus NCs under ambient and UV light. Pure $CsPbBr_3$ NCs with sizes close to those of the $CsPbBr_3$ domains in Janus NCs were prepared for comparison.

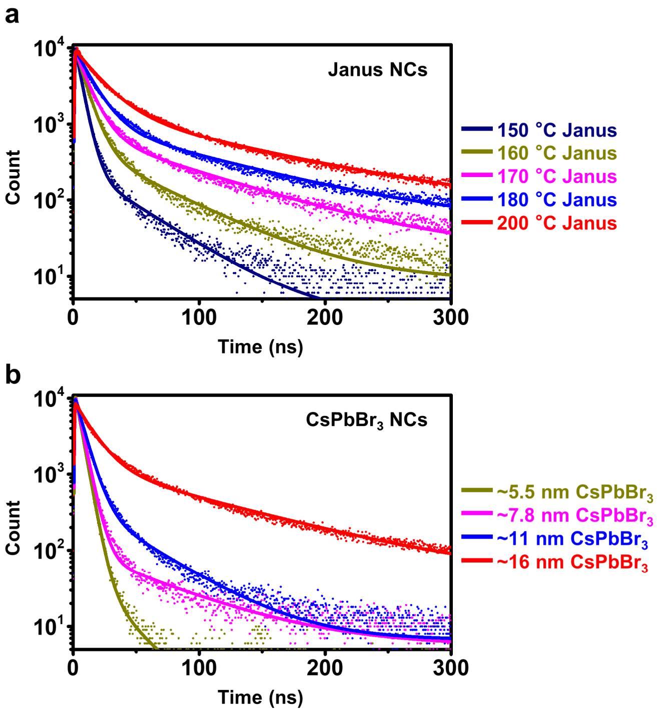

Figure S17. PL decay traces of Janus NCs and corresponding control $CsPbBr_3$ NCs. PL decay traces of (a) Janus NCs synthesized at 150–220 °C with the S–ODE and a small amount of DDT as sulfur precursors, and (b) corresponding control $CsPbBr_3$ NCs. Traces of the Janus and control NCs with similar edge lengths of $CsPbBr_3$ are labeled with same color.

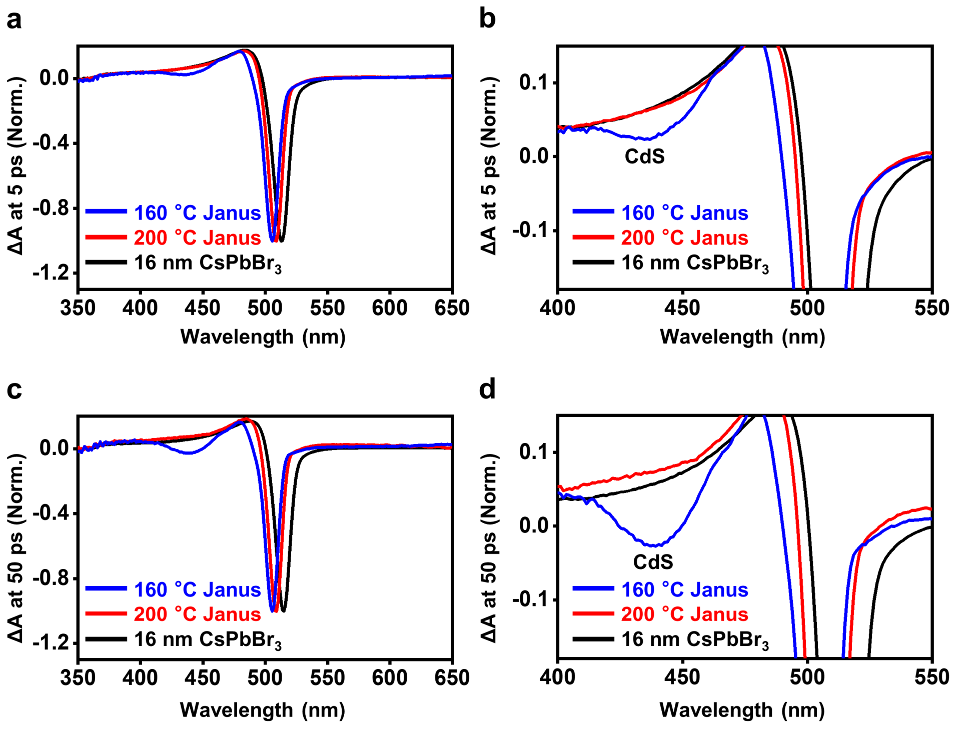

Figure S18. Additional TA spectra of Janus NCs collected with a pump wavelength of 510 nm. (a,b) TA spectra collected at 5 ps and (c,d) TA spectra collected at 50 ps. Features related to exciton bleach of CdS (at ~440 nm) were observed in Janus NCs synthesized at 160 °C as soon as 5 ps. The CdS exciton bleach formation also occurred (at ~455 nm) for Janus NCs synthesized at 200 °C, albeit the lower intensities at 5 or 50 ps.

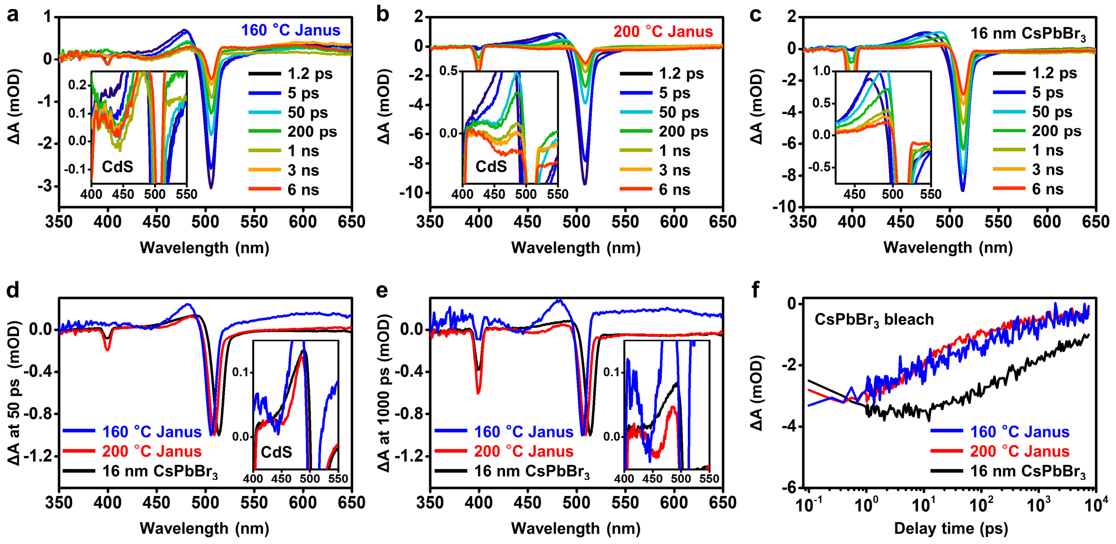

Figure S19. TA spectra of Janus NCs collected with a pump wavelength of 400 nm. (a–c) TA spectra of Janus NCs synthesized at (a) 160 and (b) 200 °C and (c) 16 nm $CsPbBr_3$ NCs, at a pump wavelength of 400 nm. (d,e) Comparison of TA spectra of these samples at 50 and 1000 ps, respectively, highlighting the bleach of CdS excitons in Janus NCs (insets in (d,e)). (f) The TA kinetics of samples in the bleach related to $CsPbBr_3$ excitons.

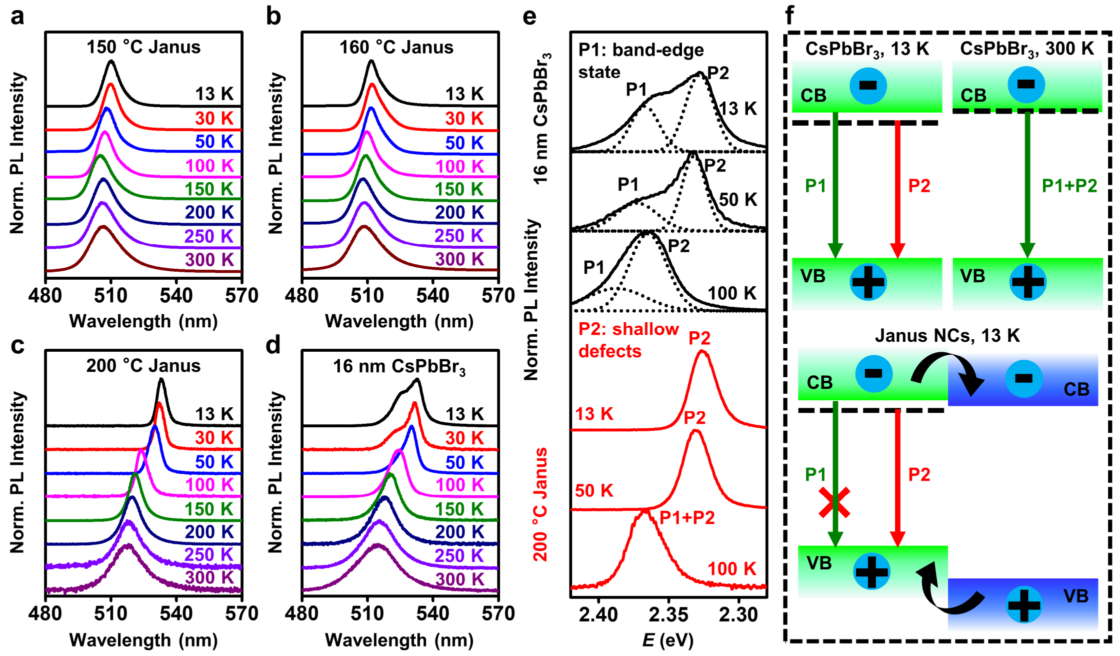

Figure S20. Temperature-dependent PL spectra of Janus NCs. (a–d) Temperature-dependent PL spectra of thin films of Janus NCs synthesized at (a) 150, (b) 160, (c) 200 °C and (d) 16 nm $CsPbBr_3$ NCs. (e) Devoluted PL peaks of 16 nm $CsPbBr_3$ NCs (top) and Janus NCs synthesized at 200 °C (bottom). PL spectra of $CsPbBr_3$ NC films showed contributions from both P1 (band edge) and P2 (shallow states) below 100 K, while the spectra of Janus NCs were mostly symmetric. (f) Tentative scheme shows the origin of P1 and P2 pathways in $CsPbBr_3$ NCs and the absence of P1 pathways in Janus NCs due to electron transfer. Smaller Janus NCs (a,b) show smaller blue shifts compared to larger Janus NCs (c) at increased temperatures. This trend was also observed in $CsPbBr_3$ NCs with different sizes in previous reports.[20]

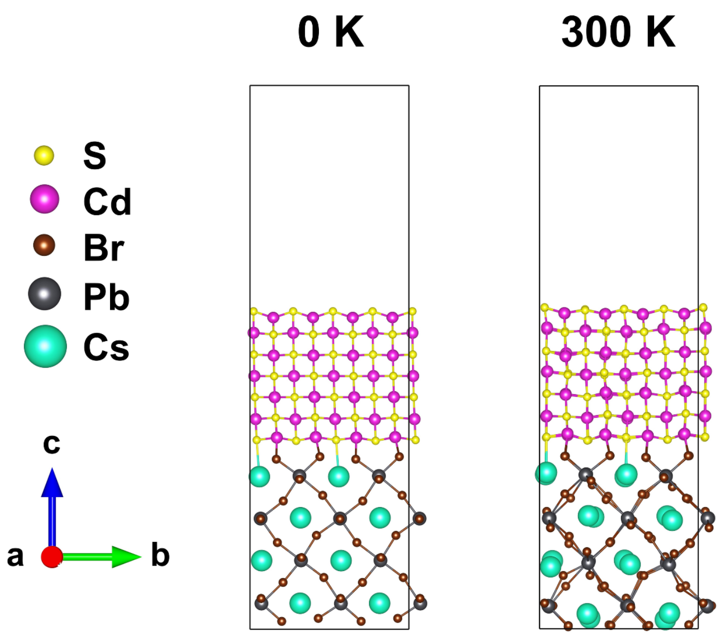

Figure S21. Optimized geometry of the $CsPbBr_3$/CdS heterojunction and a representative snapshot during MD simulation at 300 K. The heterojunction is formed by interfacing the CsBr-terminated $CsPbBr_3$ (110) surface with the CdS (220) surface. The calculated average Cs–S and Br–Cd bond lengths in the optimized geometry are 3.733 and 3.053 Å, which decrease to 3.626 and 2.714 Å at 300 K, respectively. Therefore, thermal fluctuations enhance the interfacial interaction and favor electron transfer.

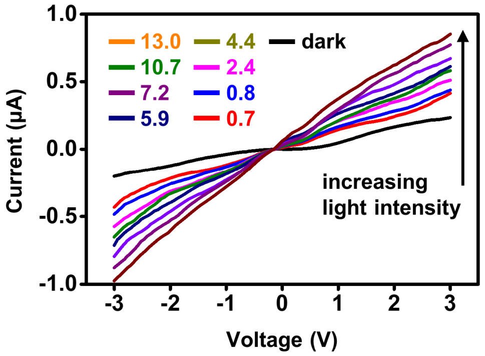

Figure S22. *I–V* characteristics of thin-film photoconductors made from 16 nm $CsPbBr_3$ NCs under different light intensities.

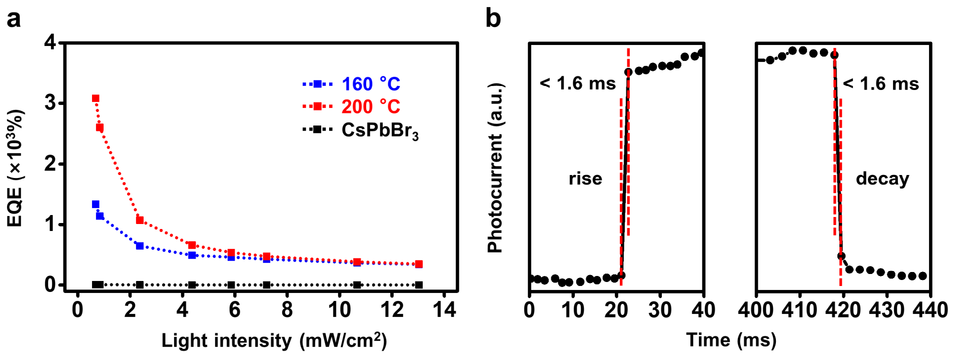

Figure S23. EQE and response time of the photoconductors. (a) EQEs of devices made from Janus NCs synthesized at 160 and 200 °C and also bare $CsPbBr_3$ NCs at various light intensities. Bias, 3.0 V. The highest EQEs for devices were measured at 0.7 mW cm^{-2}, and were 1334.3%, 3082.2% and 7.3% for 160 °C Janus NCs, 200 °C Janus NCs, and 16 nm $CsPbBr_3$ NCs, respectively. (b) The rise and decay responses of devices made from Janus NCs synthesized at 160 °C. Note 1.6 ms was the smallest time interval for data collection in the measurements, limited by our instrument. Thus, the actual response time should be smaller than 1.6 ms.

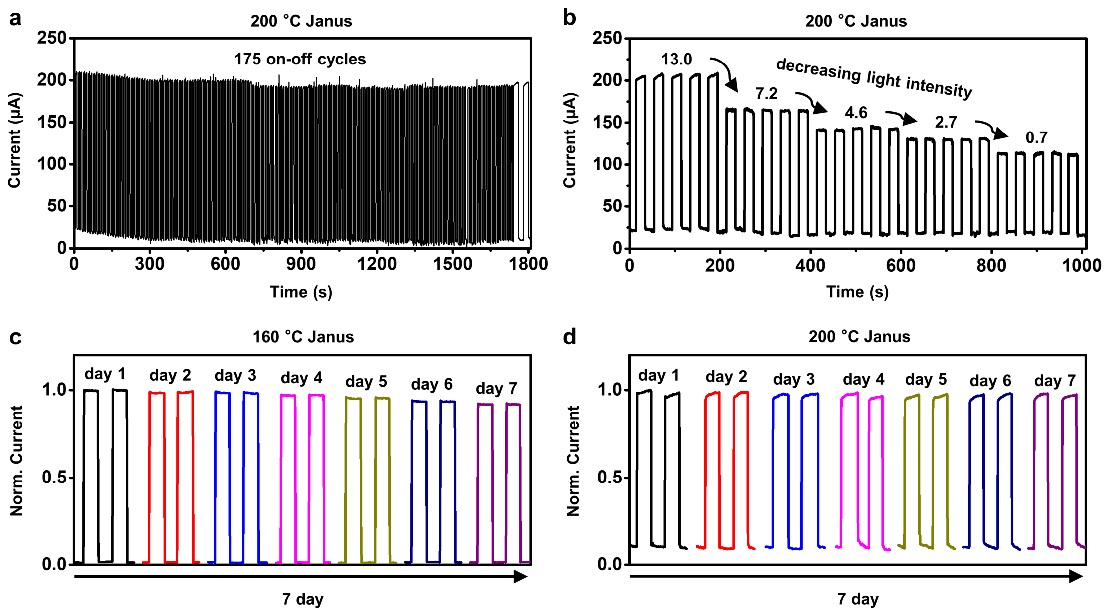

Figure S24. Stability of Janus NC based photoconductors. (a,b) The photocurrents of device made from Janus NCs (synthesized at 200 °C) under continuous illumination test under ambient atompsphere by using (a) constant light intensity (13.0 mW cm^{-2}) for 175 on-off cycles (~30 min) or (b) various light intensities. (c,d) The changes in photocurrents of devices base on Janus NCs synthesized at (c) 160 and (d) 200 °C after stored under ambient atmosphere (room temperature of ~20 °C and humidity of ~35%) for 7 days. For (c,d), the photocurrents were normalized by the photocurrent measured on day 1. The relative changes in photocurrents were measured on each day. All the on-off switching behavior of devices in (a,b,c,d) were measured under a light intensity of 13.0 mW cm^{-2} and a bias of 3.0 V.

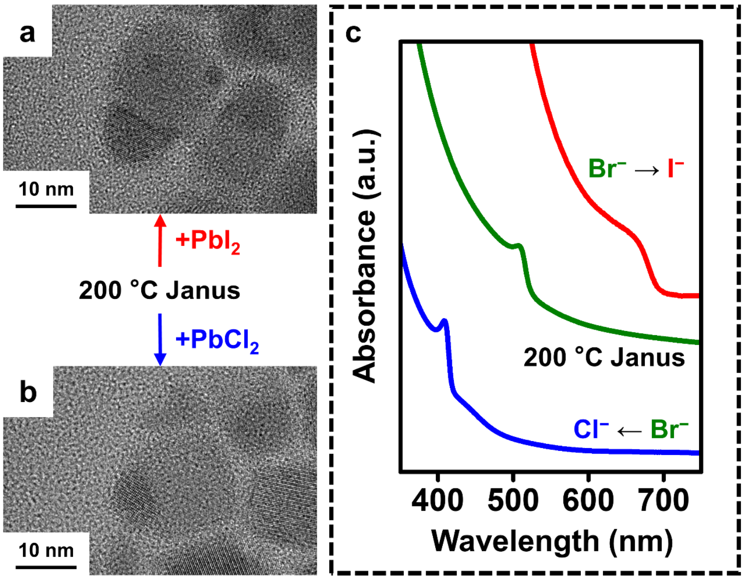

Figure S25. $CsPbX_3$/CdS (X = Cl, I) Janus NCs. (a,b) TEM images of $CsPbI_3$/CdS and $CsPbCl_3$/CdS Janus NCs via anion exchange. (c) UV-visible absorption spectra of Janus NCs with different cesium lead halide domains.

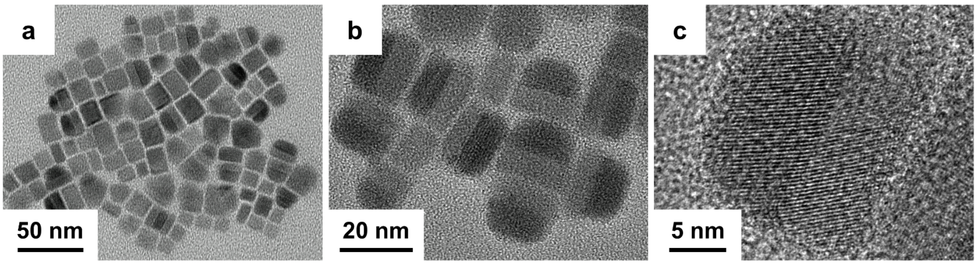

Figure S26. TEM image of $CsPbBr_3$/ZnS Janus NCs.

Table S1. Summary of PL peak position of Janus NCs and corresponding control $CsPbBr_3$ NCs.

**PL Peak of Janus NCs (nm)**	**PL Peak of $CsPbBr_3$ NCs (nm)**
504 (150 °C Janus)	/
507 (160 °C Janus)	492 (~5.5 nm)
510 (170 °C Janus)	498 (~7.6 nm)
513 (180 °C Janus)	515 (~11 nm)
515 (200 °C Janus)	516 (~16 nm)
516 (220 °C Janus)	/

Table S2. Fitting data of the PL decay curves of Janus NCs and corresponding control $CsPbBr_3$ NCs in Figure S17. Data for Janus NCs and control samples with similar sizes in $CsPbBr_3$ domains are in the same color shades.

Samples	λ/nm	τ_1 (B1%)/ns	τ_2 (B2%)/ns	τ_{ave}/ns
**150 °C Janus**	504	4.90 (83.17%)	40.70 (16.83%)	5.75
**160 °C Janus**	507	7.60 (73.60%)	51.92 (26.40%)	9.81
**~5.5 nm $CsPbBr_3$**	492	4.64 (97.14%)	20.65 (2.86%)	4.75
**170 °C Janus**	510	9.80 (59.27%)	78.66 (40.73%)	15.23
**~7.6 nm $CsPbBr_3$**	489	5.31 (91.34%)	62.25 (8.66%)	5.77
**180 °C Janus**	513	12.60 (54.14%)	92.19 (45.86%)	20.86
**~11 nm $CsPbBr_3$**	515	6.98 (79.94%)	41.60 (20.06%)	8.38
**200 °C Janus**	515	18.27 (47.66%)	105.89 (52.34%)	32.23
**~16 nm $CsPbBr_3$**	516	13.25 (45.50%)	93.07 (54.50%)	24.88

B1 and B2 are parameters in the bi-exponential fitting of the time-resolved PL and presented in the form of weighted percentage of the two decays. The fitting follows $y = A + B_1 e^{(-\frac{x}{\tau_1})} + B_2 e^{(-\frac{x}{\tau_2})}$. The average PL lifetime (τ_{ave}) was provided by the commercial software for the TCSPC toolkit on Horiba FluoroMax Plus spectrometer.

Table S3. The temperature-dependent PL peak position and width (FWHM) of Janus NCs.

T (K)	150 °C Janus		160 °C Janus		200 °C Janus	
	E_P (eV)	FWHM (eV)	E_P (eV)	FWHM (eV)	E_P (eV)	FWHM (eV)
**13**	2.43059	0.04072	2.42421	0.03878	2.32957	0.02235
**30**	2.43218	0.04157	2.42183	0.04500	2.33454	0.02312
**50**	2.44182	0.04666	2.42342	0.04432	2.34251	0.02302
**100**	2.44424	0.04679	2.43298	0.04551	2.37044	0.03037
**150**	2.45642	0.05872	2.43378	0.05119	2.38339	0.03461
**200**	2.45805	0.05903	2.44101	0.06029	2.38972	0.04876
**250**	2.45969	0.06795	2.44343	0.07048	2.39640	0.06533
**300**	2.46050	0.07991	2.44586	0.08325	2.40161	0.07975

Table S4. The temperature-dependent PL peak position and width (FWHM) of $CsPbBr_3$ NCs.

	**16 nm $CsPbBr_3$**			
**T (K)**	**E_{P1} (eV)**	**FWHM1 (eV)**	**E_{P2} (eV)**	**FWHM2 (eV)**
**13**	2.36702	0.02448	2.32799	0.03909
**30**	2.37280	0.03622	2.33203	0.01891
**50**	2.37433	0.04871	2.33811	0.02194
**100**	2.38732	0.04898	2.36480	0.03128
**150**	2.38871	0.04922		
**200**	2.39704	0.06022		
**250**	2.40642	0.08168		
**300**	2.40945	0.10212		

Table S5. Summary of device performance of photoconductors made from Janus NCs and $CsPbBr_3$ NCs. Wavelength, 450 nm, bias = 3.0 V. D^* in Table S5 was measured by using I_{dark}. For the listed examples, D^* was measured by using Idark, which may be overestimated by 2–3 orders of magnitude compared to those obtained from Flicker noise.

**Samples**	***P* (mW/cm^2)**	**I_{ligh}/I_{dark}**	***R* (A/W)**	***D* (10^{13} Jones)**
**160 °C**	13.0	5100	1.3	1.2
**Janus**	0.7	1022	4.8	4.8
**200 °C**	13.0	9.7	1.3	0.05
**Janus**	0.7	5.0	11.2	0.45
**16 nm**	13.0	3.6	0.005	0.002
**$CsPbBr_3$**	0.7	1.8	0.026	0.01

Table S6. Comparison of device performance of reported photoconductors involving perovskite heterostructures or additional charge transport layers. For the listed examples, D^* were measured by using I_{dark}, except the one shown in ref. (28). The measurements by using I_{dark} may provide overestimated D^*, by 2–3 orders of magnitude, compared to those obtained from Flicker noise.

Materials	λ (nm)	Bias (V)	I_{ligh}/I_{dark}	R (A/W)	D (Jones)	Ref.
$CsPbI_{3-x}Br_x$ nanowires/2D MoS_2	355	1	/	3.7×10^3	1.9×10^{12}	[21]
$CsPbBr_3$ nanowires/conjugated polymer transport layer	350	0.1	/	0.25	1.2×10^{13}	[22]
$MAPbI_3$ nanofilm/O-doped CdS nanorod array	700	0	750	0.48	2.1×10^{13}	[23]
$CsPbBr_3$ QDs/ZnO/glassy-graphene	405	1	/	4.0×10^{-4}	/	[24]
2D $CsPbBr_3$/CdS flakes	500	10	$\approx 10^5$	0.49	3.7×10^{10}	[25]
2D/3D (4-AMP)$(MA)_2Pb_3Br_{10}$/$MAPbBr_3$	405	0	$\approx 10^5$	1.2×10^{-3}	1.3×10^{12}	[26]
$CsPbBr_3$ NCs/2D CdS_xSe_{1-x}	405	3	1.36×10^5	2.9×10^2	1.3×10^{14}	[27]
$CsPbBr_3$ QDs/organic semiconductor transport layer	450	3	/	1.6×10^4	3.2×10^{12}	[28]
**Epitaxial $CsPbBr_3$/CdS Janus NCs**	**450**	**3**	**5×10^3**	**4.83**	**4.8×10^{13}**	**This work**

References

[1] L. Protesescu, S. Yakunin, M. I. Bodnarchuk, F. Krieg, R. Caputo, C. H. Hendon, R. X. Yang, A. Walsh, M. V. Kovalenko, *Nano Lett.* **2015**, *15*, 3692.

[2] L. Peng, A. Dutta, R. Xie, W. Yang, N. Pradhan, *ACS Energy Lett.* **2018**, *3*, 2014.

[3] H. Zhang, J. Jang, W. Liu, D. V. Talapin, *ACS Nano* **2014**, *8*, 7359.

[4] X. Luo, G. Liang, Y. Han, Y. Li, T. Ding, S. He, X. Liu, K. Wu, *J. Am. Chem. Soc.* **2020**, *142*, 11270.

[5] E. Deligoz, K. Colakoglu, Y. Ciftci, *Physica B: Condensed Matter* **2006**, *373*, 124.

[6] S. Ouendadji, S. Ghemid, H. Meradji, F. E. H. Hassan, *Comp. Mater. Sci.* **2011**, *50*, 1460.

[7] B. J. Schwartz, E. R. Bittner, O. V. Prezhdo, P. J. Rossky, *J. Chem. Phys.* **1996**, *104*, 5942.

[8] B. F. Habenicht, O. V. Prezhdo, *Phys. Rev. Lett.* **2008**, *100*, 197402.

[9] J. C. Tully, *J. Chem. Phys.* **1990**, *93*, 1061.

[10] C. F. Craig, W. R. Duncan, O. V. Prezhdo, *Phys. Rev. Lett.* **2005**, *95*, 163001.

[11] S. A. Fischer, B. F. Habenicht, A. B. Madrid, W. R. Duncan, O. V. Prezhdo, *J. Chem. Phys.* **2011**, *134*, 024102.

[12] A. V. Akimov, O. V. Prezhdo, *J. Chem. Theory Comput.* **2014**, *10*, 789.

[13] A. V. Akimov, O. V. Prezhdo, *J. Chem. Theory Comput.* **2013**, *9*, 4959.

[14] G. Kresse, J. Furthmüller, *Phys. Rev. B* **1996**, *54*, 11169.

[15] J. P. Perdew, K. Burke, M. Ernzerhof, *Phys. Rev. Lett.* **1996**, *77*, 3865.

[16] P. E. Blöchl, *Phys. Rev. B* **1994**, *50*, 17953.

[17] H. J. Monkhorst, J. D. Pack, *Phys. Rev. B* **1976**, *13*, 5188.

[18] S. Grimme, J. Antony, S. Ehrlich, H. Krieg, *J. Chem. Phys.* **2010**, *132*, 154104.

[19] S. Grimme, S. Ehrlich, L. Goerigk, *J. Comput. Chem.* **2011**, *32*, 1456.

[20] P. Ijaz, M. Imran, M. M. Soares, H. C. Tolentino, B. Martín-García, C. Giannini, I. Moreels, L. Manna, R. Krahne, *J. Phys. Chem. Lett.* **2020**, *11*, 2079.

[21] H. Wu, Z. Kang, Z. Zhang, Z. Zhang, H. Si, Q. Liao, S. Zhang, J. Wu, X. Zhang, Y. Zhang, *Adv. Funct. Mater.* **2018**, *28*, 1802015.

[22] F. Cao, W. Tian, K. Deng, M. Wang, L. Li, *Adv. Funct. Mater.* **2019**, *29*, 1906756.

[23] F. Cao, L. Meng, M. Wang, W. Tian, L. Li, *Adv. Mater.* **2019**, *31*, 1806725.

[24] K. Shen, X. Li, H. Xu, M. Wang, X. Dai, J. Guo, T. Zhang, S. Li, G. Zou, K.-L. Choy, *J. Mater. Chem. A* **2019**, *7*, 6134−6142.

[25] B. Jin, N. Zuo, Z.-Y. Hu, W. Cui, R. Wang, G. Van Tendeloo, X. Zhou, T. Zhai, *Adv. Funct. Mater.* **2020**, *30*, 2006166.

[26] X. Zhang, C. Ji, X. Liu, S. Wang, L. Li, Y. Peng, Y. Yao, M. Hong, J. Luo, *Adv. Optical Mater.* **2020**, *8*, 2000311.

[27] M. Peng, Y. Ma, L. Zhang, S. Cong, X. Hong, Y. Gu, Y. Kuang, Y. Liu, Z. Wen, X. Sun, *Adv. Funct. Mater.* **2021**, *31*, 2105051.

[28] K. Chen, X. Zhang, P. A. Chen, J. Guo, M. He, Y. Chen, X. Qiu, Y. Liu, H. Chen, Z. Zeng, *Adv. Sci.* **2022**, 2105856.
